# The chromatin remodeler ERCC6 and the histone chaperone NAP1 are involved in apurinic/apyrimidinic endonuclease-mediated DNA repair

**DOI:** 10.1093/plcell/koae052

**Published:** 2024-02-17

**Authors:** Tianyi Fan, Tianfang Shi, Ran Sui, Jingqi Wang, Huijia Kang, Yao Yu, Yan Zhu

**Affiliations:** State Key Laboratory of Genetic Engineering, Collaborative Innovation Center for Genetics and Development, School of Life Sciences, Fudan University, Shanghai 200438, China; State Key Laboratory of Genetic Engineering, Collaborative Innovation Center for Genetics and Development, School of Life Sciences, Fudan University, Shanghai 200438, China; Shanghai Engineering Research Center of Industrial Microorganisms, Shanghai 200438, China; State Key Laboratory of Genetic Engineering, Collaborative Innovation Center for Genetics and Development, School of Life Sciences, Fudan University, Shanghai 200438, China; State Key Laboratory of Genetic Engineering, Collaborative Innovation Center for Genetics and Development, School of Life Sciences, Fudan University, Shanghai 200438, China; Shanghai Engineering Research Center of Industrial Microorganisms, Shanghai 200438, China; State Key Laboratory of Genetic Engineering, Collaborative Innovation Center for Genetics and Development, School of Life Sciences, Fudan University, Shanghai 200438, China; Department of Horticulture, Zhejiang University, Hangzhou 310058, China; State Key Laboratory of Genetic Engineering, Collaborative Innovation Center for Genetics and Development, School of Life Sciences, Fudan University, Shanghai 200438, China; Shanghai Engineering Research Center of Industrial Microorganisms, Shanghai 200438, China; State Key Laboratory of Genetic Engineering, Collaborative Innovation Center for Genetics and Development, School of Life Sciences, Fudan University, Shanghai 200438, China

## Abstract

During base excision repair (BER), the apurinic or apyrimidinic (AP) site serves as an intermediate product following base excision. In plants, APE-redox protein (ARP) represents the major AP site of cleavage activity. Despite the well-established understanding that the nucleosomal structure acts as a barrier to various DNA-templated processes, the regulatory mechanisms underlying BER at the chromatin level remain elusive, especially in plants. In this study, we identified plant chromatin remodeler Excision Repair Cross-Complementing protein group 6 (ERCC6) and histone chaperone Nucleosome Assembly Protein 1 (NAP1) as interacting proteins with ARP. The catalytic ATPase domain of ERCC6 facilitates its interaction with both ARP and NAP1. Additionally, ERCC6 and NAP1 synergistically contribute to nucleosome sliding and exposure of hindered endonuclease cleavage sites. Loss-of-function mutations in Arabidopsis (*Arabidopsis thaliana*) *ERCC6* or *NAP1* resulted in *arp*-dependent plant hypersensitivity to 5-fluorouracil, a toxic agent inducing BER, and the accumulation of AP sites. Furthermore, similar protein interactions are also found in yeast cells, suggesting a conserved recruitment mechanism employed by the AP endonuclease to overcome chromatin barriers during BER progression.

## Teaser

APURINIC/APYRIMIDINIC ENDONUCLEASE REDOX PROTEIN recruits a chromatin remodeler and a histone chaperone to facilitate uracil N-glycosylase-triggered base excision repair.

**IN A NUTSHELL**

**Background:** DNA bases are susceptible to damage from environmental factors such as UV light or reactive oxygen species. Base excision repair (BER) can eliminate modified or damaged DNA bases. However, chromatin structures pose natural obstacles to the recognition and repair of DNA lesions. The mechanisms governing BER within the context of chromatin are imperative for maintaining genome integrity. However, the factors or mechanisms involved in facilitating chromatin mobility to aid BER remain poorly elucidated, particularly in plants, which experience extensive DNA base damage due to their stationary lifestyle and oxidative stress associated with photosynthesis.

**Question:** APURINIC/APYRIMIDINIC ENDONUCLEASE REDOX PROTEIN (ARP) is the predominant enzyme responsible for cleaving apurinic/apyrimidinic sites in plants. Given its preferential endonuclease activity toward naked DNA, the question arises as to which chromatin-related proteins can be specifically recruited by ARP to enhance local chromatin mobility and facilitate efficient BER in plants.

**Findings:** We identified the plant chromatin remodeler ERCC6 and histone chaperone NAP1 as interacting partners with ARP. In a synergistic manner, ERCC6 and NAP1 contribute to the sliding of nucleosomes and exposure of hindered endonuclease cleavage sites. Loss-of-function mutations in Arabidopsis *ERCC6* or *NAP1* resulted in an *arp*-dependent hypersensitivity of plants to a toxic agent that induces BER, leading to the accumulation of apurinic or apyrimidinic sites. Furthermore, we showed that these proteins also interact with each other in yeast cells, suggesting a conserved recruitment mechanism employed by the apurinic or apyrimidinic endonuclease to overcome chromatin barriers during BER progression.

**Next Steps:** Future biochemical and molecular analyses will be imperative to unravel the intricate recruitment mechanisms of specific chromatin factors in distinct BER pathways at the chromatin level. This will advance our comprehension of the co-evolutionary dynamics between epigenetic machineries, their regulatory mechanisms, and chromatin architecture across diverse organisms for genome maintenance.

## Introduction

DNA bases exhibit limited chemical stability and are susceptible to alteration. Base excision repair (BER) is the critical DNA repair mechanism for removing modified or damaged DNA bases, which are nonhelix-distorting lesions caused primarily by oxidation, deamination, hydrolysis, or alkylation (Krokan and Bjoras 2013; Menoni et al. 2017). As the first step of BER, specific DNA glycosylases selectively target their corresponding base substrates and cleave the C1′–N-glycosylic bond, leading to the formation of either an apurinic or apyrimidinic (AP) site. These AP sites act as obstacles for replicative polymerases due to loss of coding information. Moreover, they possess cytotoxic and carcinogenic properties by inducing abnormal DNA–protein crosslinks. Some DNA glycosylases act as bifunctional glycosylases/lyases by additionally cleaving the deoxyribose-phosphate backbone through their DNA lyase activity subsequent to base removal (Akishev et al. 2016). In cases involving monofunctional glycosylase activity, AP sites are recognized by AP endonucleases (APEs), which hydrolyze them at their 5′ side resulting in a gapped single-strand break with 3′-OH and 5′-deoxyribose-5-phosphate terminus. BER proceeds gap-filling synthesis performed by DNA polymerases and nick seal performed by DNA ligases, respectively (Cordoba-Canero et al. 2009; Krokan and Bjoras 2013; Menoni et al. 2017; Hindi et al. 2021).

5-Fluorouracil (5-FU), an analog of uracil, is extensively utilized as a chemotherapeutic agent for cancer treatment to inhibit thymidylate synthase (Wyatt and Wilson 2009). It depletes thymidine nucleotides required for DNA synthesis and promotes the accumulation of uracil residues in DNA. Uracil residues in DNA can also arise from cytosine deamination, which is the most common spontaneous base modification, resulting in the formation of premutagenic U•G mispairs (Vertessy and Toth 2009). Uracil DNA glycosylase (UDG) is a monofunctional glycosylase that specifically recognizes uracil within DNA. It efficiently excises uracil base and generates an AP site as the reaction product (Wyatt and Wilson 2009; Cordoba-Canero et al. 2010; Roldan-Arjona et al. 2019). In Arabidopsis (*Arabidopsis thaliana*), Uracil N-glycosylase (UNG) encodes the primary UDG activity, and *ung* null mutant exhibits remarkable tolerance to 5-FU treatment along with increased uracil accumulation in DNA (Cordoba-Canero et al. 2010). Meanwhile, APE-redox protein (ARP) serves as the predominant enzyme responsible for cleaving AP sites in plants, despite the existence of its two sequence-related homologues known as AP endonuclease 1-like (APE1L) and APE2 (Cordoba-Canero et al. 2009, 2011; Li et al. 2023). Although displaying no phenotypic defects under normal growth conditions, the ARP-depleting mutant shows hypersensitivity to 5-FU, indicative of the critical role of ARP in resolving AP lesions, the intermediates generated by the monofunctional UNG (Cordoba-Canero et al. 2011).

In eukaryotic cells, DNA strands are compacted into multiorder chromatin structures that present natural barriers to DNA lesion recognition and repair. Nucleosomes generally reduce DNA mobility and maintain local structural features that limit protein–DNA interplay. ATP-dependent Snf2-type chromatin remodelers can modulate the histone–DNA interactions by using the energy released from ATP hydrolysis, and thereby facilitate nucleosome sliding, relocating, removing, or intrinsic component exchanging (Clapier and Cairns 2009; Clapier et al. 2017). Histone chaperones are small proteins with high affinity to distinct histones, and are required in nucleosome assembly/disassembly processes by preventing nonspecific histone–DNA interactions (Zhou et al. 2015). Chromatin remodelers and histone chaperones have been reported to act alone or synergistically depending on the relevant chromatin events to modulate the local or global structure in diverse biological processes (Kang et al. 2022a). It was reported that the activities of human UDG and APE, key players in the early stage of BER, were greatly reduced in the presence of histones on nucleosomal DNA (Nilsen et al. 2002; Beard et al. 2003; Hinz 2014). BER machinery for 8-oxoguanine (8-oxoG), a very common base lesion induced by oxidation, was strongly impeded in nucleosome core DNA in vitro, which required additional chromatin remodeler to stimulate BER efficiency (Menoni et al. 2007). However, Nakanishi et al. (2007) reported that human UDG and APE act efficiently on highly folded oligo-nucleosome arrays without requiring substantial disruption, and chromatin remodeling activity is only needed in the latter steps of BER. Without the support of further genetic evidence, these conflicting in vitro findings cannot fully reflect the complexity of the BER that occurs in the context of chromatin in vivo. Compared with animals, a high amount of DNA base damage occurs in plants during growth and development, which may be partially due to their sessile lifestyle and the oxidative stress accompanied with photosynthesis (Nisa et al. 2019). Therefore, the regulatory mechanisms of BER in the plant chromatin context are crucial for plant genome integrity. However, they are still poorly understood.

Cockayne syndrome (CS) is a rare autosomal recessive genetic disorder that belongs to the segmental premature aging syndrome. Approximately 80% of patients suffering from CS are classified as CS complementation group B (CSB), and they carry mutations in the *EXCISION REPAIR CROSS-COMPLEMENTING PROTEIN GROUP 6* (*ERCC6*) gene (hereafter named as mammalian *ERCC6^CSB^*) (Licht et al. 2003). As a member of Snf2-type chromatin remodelers involved in global chromatin maintenance (Newman et al. 2006), ERCC6^CSB^ is best known in its interaction with RNA polymerase II, and numerous studies have implicated human ERCC6^CSB^ and its yeast homolog Rad26 in transcription-coupled nucleotide excision repair (TC-NER) (Troelstra et al. 1992; van der Horst et al. 1997; van den Boom et al. 2004; Duan et al. 2020; Xu et al. 2020). Both CHR8 and CHR24 are considered as Arabidopsis homologs of ERCC6 (Shaked et al. 2006; Knizewski et al. 2008); however, only one early study on selected plant remodelers has revealed that mutation in *CHR24* is sensitive to γ-irradiation and UV-C (Shaked et al. 2006). No further biochemical and molecular characterization of these two remodelers has been carried out. Nucleosome Assembly Protein 1 (NAP1) proteins are highly conserved histone chaperones with affinity to H2A/H2B core histones (Zhou et al. 2015). Three Arabidopsis NAP1 homologs (NAP1;1 to NAP1;3) form a chaperone family with high functional redundancy, and are involved in transcriptional regulation, chromatin stability, and NER and homologous recombination (Liu et al. 2009; Gao et al. 2012).

In this study, we identified Arabidopsis ERCC6 and NAP1 homologs as ARP-interacting proteins. ERCC6 and NAP1 exhibit synergistic activity in nucleosome mobilization and play a crucial role in ARP-mediated AP elimination, thereby enhancing plant tolerance to 5-FU. These findings suggest that ERCC6 and NAP1 are involved in the integral machinery facilitating the progression of BER.

## Results

### Mutual protein interaction among Arabidopsis ARP–CHR24–NAP1

We expressed Arabidopsis ARP as a recombinant protein tagged with maltose-binding protein (MBP) and confirmed its endonuclease activity in vitro. MBP–ARP exhibited intrinsic AP endonuclease activity by cleaving a DNA duplex containing tetrahydrofuran (THF, a synthetic analog of an abasic site)•G pair but not the control C•G pair (Supplementary Fig. S1A). By using MBP–ARP as bait, we performed pulldown assays followed by Mass Spectrum (pulldown-MS) analysis to identify interacting proteins from Arabidopsis seedlings. In this study, we identified histone chaperone NAP1;1 and NAP1;2 and chromatin remodeler CHR24 as ARP-interacting proteins (Supplementary Data Set 1).

**Figure 1.**
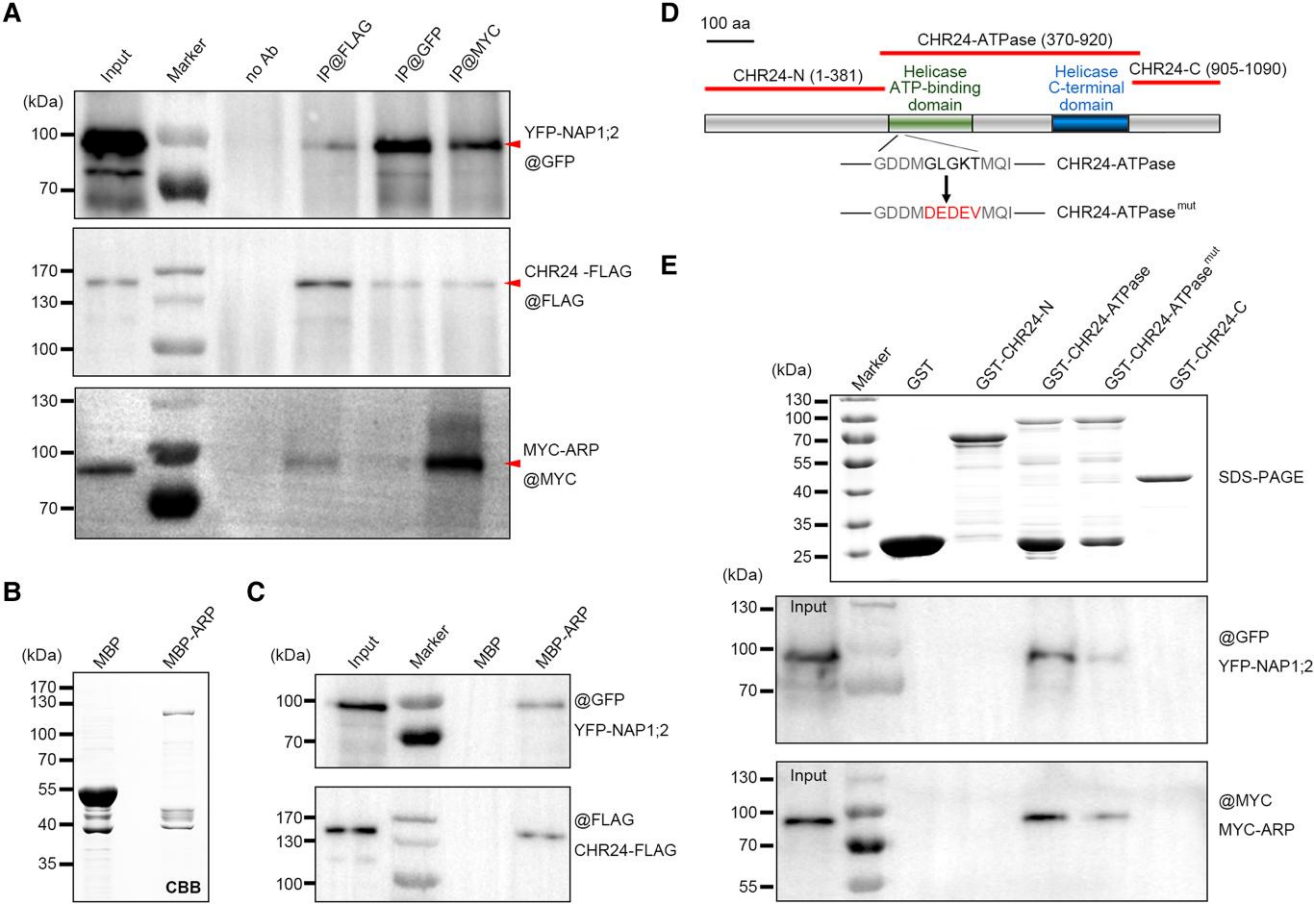
ARP interacts with chromatin remodeler CHR24 and histone chaperone NAP1 proteins. **A)** co-IP was performed to detect the interactions between YFP-NAP1;2, CHR24-FLAG, and MYC-ARP in planta. Total protein extracts from the mesophyll protoplasts expressing tagged proteins were first immune-precipitated with or without antibodies against GFP, FLAG, and MYC, respectively, followed by immunoblot analysis of resulting fractions. The arrowheads indicate the target protein bands, taking into consideration potential protein degradation or nonspecific bands observed in the immunoblot results. **B)** Purified MBP and MBP–ARP proteins in Coomassie Brilliant Blue (CBB)-stained SDS–PAGE gel. **C)** Immobilized MBP–ARP and MBP (control) were mixed with protein extracts from mesophyll protoplasts expressing YFP-NAP1;2 and CHR24-FLAG for binding assays. **D)** A schematic diagram of CHR24 was presented along with three truncated parts: CHR24-N, CHR24-ATPase, and CHR24-C. The replacement of GLGKT site by DEDEV was shown as CHR24-ATPase^mut^. **E)** GST and GST-tagged truncated CHR24 proteins (upper panel) were purified for pulldown assay examined by antibody against GFP (YFP-NAP1;2, middle panel) or against MYC (MYC-ARP, lower panel).

In contrast to a previous report of transgenic plants with high expression levels of YFP-tagged NAP1 proteins (Liu et al. 2009), we failed to obtain transgenic Arabidopsis alleles highly expressing CHR24 or ARP protein in this study. We speculate that this may be due to the potential toxicity when these catalytic activities are ectopically expressed during plant growth and development. Alternatively, we transiently co-expressed FLAG-tagged CHR24 (CHR24-FLAG, theoretical molecular weight (tMW): approximately 120 kDa) and MYC-tagged ARP (MYC-ARP, tMW: 60 kDa) in the mesophyll protoplasts prepared from YFP-NAP1; 2 (tMW: 69 kDa) transgenic plants using the protocol previously reported (Yoo et al. 2007). Through a co-immunoprecipitation (co-IP) experiment using these materials, we demonstrated mutual protein–protein interactions among these proteins in vivo (Fig. 1A), indicating that ARP can recruit CHR24 and NAP1. Additionally, we conducted pairwise co-IP validation of ARP, CHR24, and NAP1, and our negative controls employed in these experiments substantiated the specificity of these interactions (Supplementary Fig. S2, A to C).

Furthermore, through a pulldown assay, MBP–ARP, but not negative control MBP, specifically retrieved YFP-NAP1;2 and CHR24-FLAG (Fig. 1, B and C). To provide additional evidence for the specificity of these protein interactions, we also expressed GST-UNG (Supplementary Fig. S3A); however, both YFP-NAP1;2 and CHR24-FLAG were barely retrieved in the pulldown portions (Supplementary Fig. S3B).

Compared to ARP and NAP1 proteins, CHR24 is a larger protein with a Snf2-type ATPase domain that includes both helicase ATP-binding domain and helicase C-terminal domain (Knizewski et al. 2008). To dissect the domain(s) of CHR24 mediating its interaction with ARP and NAP1, we truncated CHR24 and expressed its intact ATPase domain (370 to 920 aa), as well as the remaining N- (1 to 381 aa) and C-terminal (905 to 1,090 aa) parts as GST-tagged proteins. These are designated as CHR24-ATPase, CHR24-N, and CHR24-C, respectively (Fig. 1, D and E). In contrast to CHR24-N and CHR24-C, CHR24-ATPase can distinctly pulldown both YFP-NAP1; 2 and MYC-ARP (Fig. 1E). This suggests that this particular domain of CHR24 acts as the major interface in these protein–protein interactions.

**Figure 2.**
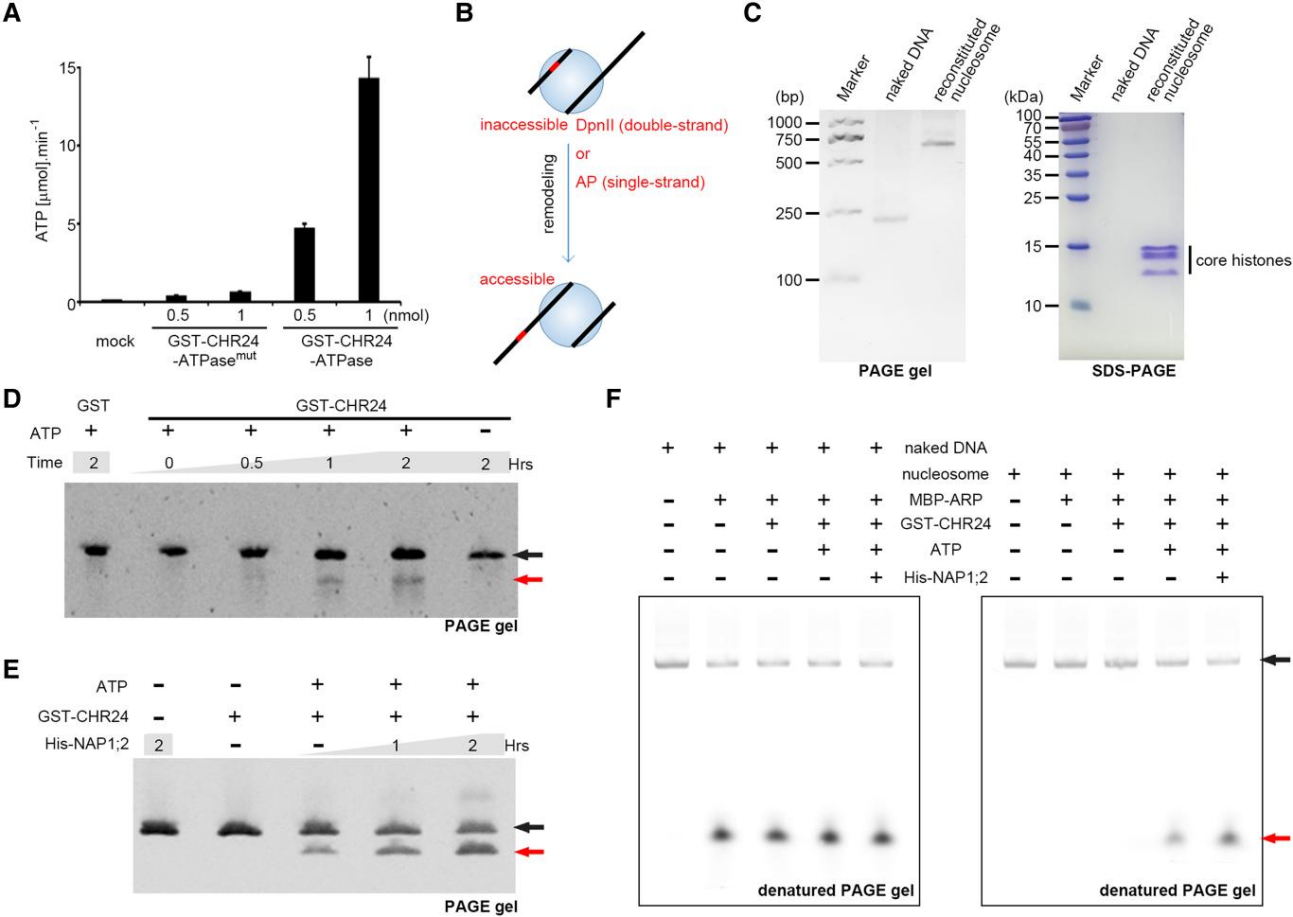
CHR24 and NAP1 can exert synergistic activity in sliding nucleosomes. **A)** The in vitro ATPase activity assay was conducted using GST-CHR24-ATPase^mut^ or GST-CHR24-ATPase protein (0.5 or 1 nmol). Mean values were shown with error bars indicating ±Sd from three biological replicate measurements. **B)** Schematic representation of nucleosome remodeling, where local nucleosome sliding exposed the inaccessible DpnII site (double-strand) or AP site (single-strand). **C)** The core histones and naked 225-bp DNA used in the nucleosome reconstitution. **D)** The ATP-dependent nucleosome remodeling was performed for indicated hours (h), with upper arrows representing intact nucleosomal DNA and the lower arrows indicating mobility of fragment cleaved by DpnII after remodeling. GST protein was used as negative control. **E)** Adding of His-NAP1; 2 greatly enhanced the remodeling activity of GST-CHR24. **F)** The ATP-dependent remodeling of AP-containing nucleosome (right panel) was performed for 1 h. AP-containing naked DNA (left panel) served as the negative control. To ensure resolution of single-stranded ARP-cleaved fragments, all DNA samples were purified and resolved in denatured PAGE gel. The intact DNA is indicated by the upper arrow, while the mobility of the fragment cleaved by ARP is highlighted by the lower arrow.

In our in vitro ATP-hydrolysis experiment, 1 nmol of GST-CHR24-ATPase protein catalyzed approximately 15 *μ*mol ATP hydrolysis per minute, indicative of the intrinsic ATPase activity of CHR24 (Fig. 2A). GXGKT (X represents any residue) is a strictly conserved site within the helicase ATP-binding domain found in many Snf2-type remodelers, where the lysine (K) residue is involved in the interaction with ATP phosphate (Bao and Shen 2007). In this study, we introduced a mutation from GLGKT to DEDEV in CHR24-ATPase, which was then designated as CHR24-ATPase^mut^ (Fig. 1, D and E). The replacement at this conserved site completely abolished the ATPase activity of CHR24 (Fig. 2A). Furthermore, compared to its native form, GST-CHR24-ATPase^mut^ exhibited reduced affinity toward both YFP-NAP1;2 and MYC-ARP proteins, suggesting that the conformational changes induced by ATP binding within the ATPase domain may, at least partially, influence the interaction of remodeler CHR24 with these associated partners (Fig. 1E). However, it cannot be entirely ruled out that these amino acid substitutions may induce substantial conformational changes in the overall protein structure.

### Molecular characterization of CHR24 and NAP1 in nucleosome sliding

We expressed full-length CHR24 tagged with GST (GST-CHR24) and NAP1;2 tagged with His (His-NAP1;2). These two proteins showed no APE activity in vitro, and also did not affect the efficiency of ARP in excising naked THF-containing DNA (Supplementary Fig. S1, A and B). We propose that this lack of activity may be attributed to their preference for nucleosomal structures over bare DNA.

We performed a chromatin remodeling assay by using reconstituted nucleosomes containing a recognition site for DpnII endonuclease (Kang et al. 2022b). The endonuclease site is originally inaccessible within the nucleosomal structure but can be exposed after remodeling (Fig. 2B). We mixed reconstituted nucleosomes (Fig. 2C) and DpnII with either GST or GST-CHR24 in the presence of ATP. After removing the proteins, we observed a smaller product of double-strand DNA cleavage in the sample mixed with GST-CHR24 which increased over time extension. In contrast, when using only negative-control GST protein or depleting ATP, no alterations were observed in the bands corresponding to nucleosomal DNA, indicating that GST-CHR24 exerts ATP-dependent chromatin remodeling activity to slide nucleosome for the exposure of endonuclease-targeting site within the nucleosomal DNA (Fig. 2D).

The histone octamer within a nucleosome consists of the central (H3/H4)_2_ tetramer and two H2A/H2B dimers at its periphery (Luger et al. 1997). We then examined the histone preference of CHR24. Intriguingly, GST-CHR24 showed specific affinity to H3/H4 histones but not H2A/H2B, even when H2A/H2B input were much overloaded in our pulldown assay (Supplementary Fig. S4). Plant NAP1 proteins have been demonstrated to preferentially bind peripheral H2A/H2B (Zhou et al. 2015). Therefore, a reasonable assumption is that NAP1 may assist remodeler CHR24 to mobilize hindered nucleosomal DNA. Indeed, His-NAP1;2 itself had no effect on endonuclease site exposure, but its addition can substantially improve the remodeling activity of GST-CHR24 (Fig. 2E).

Mechanistically, ARP catalyzes the hydrolysis of phosphodiester bonds at the 5′ side of the AP site in single-strand DNA (Balliano and Hayes 2015). We then reconstituted nucleosomes by introducing a single-stranded AP site (Fig. 2B). MBP–ARP directly recognizes the AP site on the 225 bp DNA, while the presence of nucleosome structure inhibits ARP activity. Consistent with our previous DpnII-based findings, GST-CHR24 facilitates recognition and cleavage of the nucleosome-hindered AP site in the presence of ATP, with NAP1 providing synergistic effects (Fig. 2F).

### Synergistic function of ERCC6 and NAP1 with ARP in plant resistance to base-genotoxic treatments

**Figure 3.**
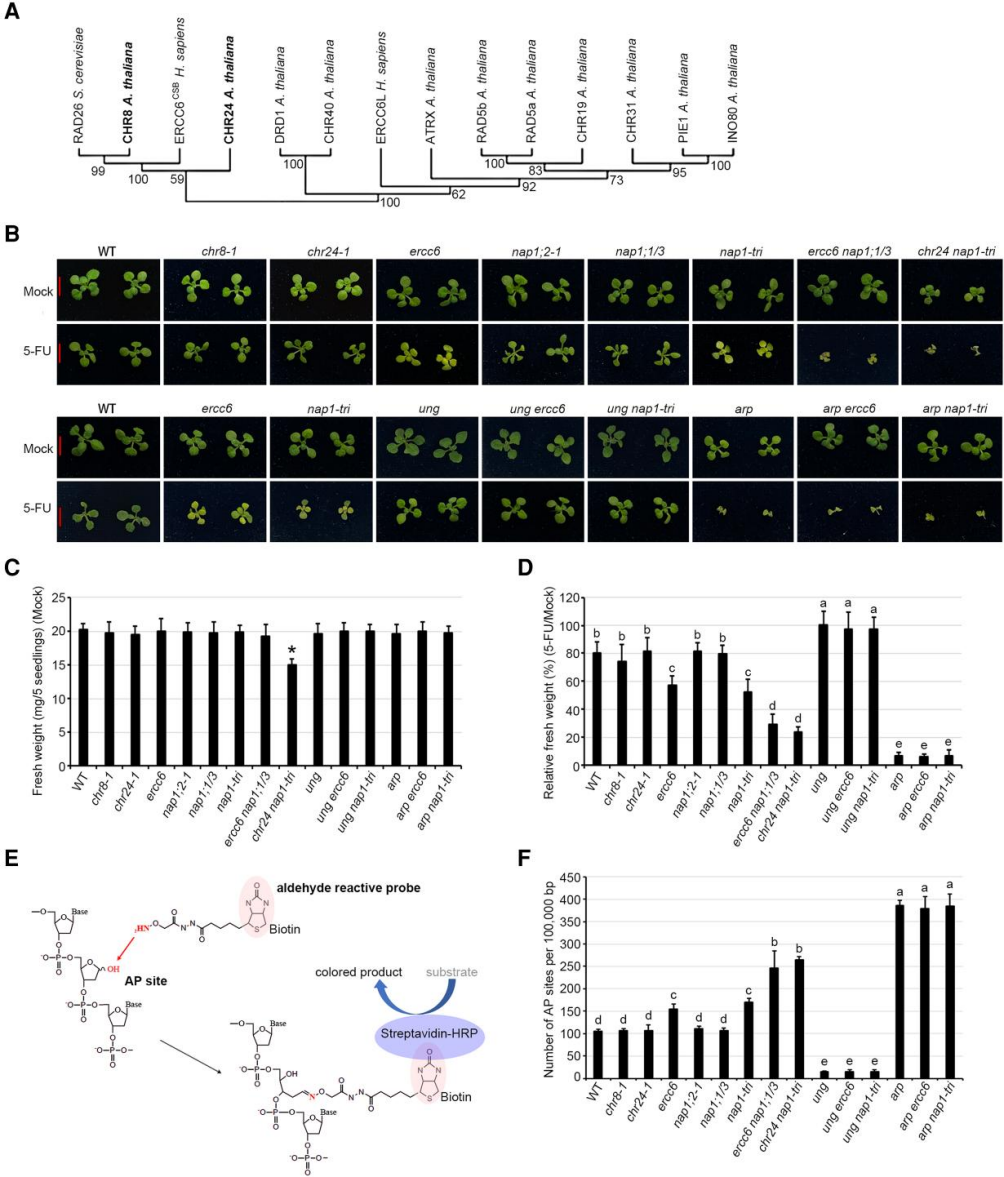
Synergistic action of ERCC6 and NAP1 members in plant resistance to 5-FU. **A)** Phylogenetic tree depicting ERCC6 members and selected plant remodelers. ERCC6 members include CHR8/24 (in bold), yeast RAD26, and human ERCC6^CSB^. Bootstrap values are indicated along branches. **B)** Images of 12-d-old plants grown on the medium with or without 5-FU. Bar = 10 mm. **C)** Fresh weight of five seedlings grown on the medium without 5-FU (Mock treatment) as one biological replicate. Mean values were shown together with error bars indicating ±Sd from 10 biologically independent replicates. Asterisks indicate significant differences between the wild type and mutant(s) (*P* < 0.05, *t*-test, two-tail). **D)** Comparison of plant sensitivities to 5-FU by normalizing fresh weights of five seedlings grown with 5-FU to those of plants grown under mock treatment conditions (as one biological replicate). Mean values were shown together with error bars indicating ±Sd from 10 biologically independent replicates. Statistically significant differences between different genotypes are denoted by distinct lowercase letters (*P* < 0.05, one-way ANOVA). **E)** Schematic diagram illustrating AP detection method using aldehyde reactive probe for biotin tagging at AP site, followed by recognition through Streptavidin-Horseradish Peroxidase (HRP) conjugate catalyzing substrate into colored product formation. **F)** Comparison of AP level in the genomic DNA extracted from plants in (**B**). Mean values were shown together with error bars indicating ±Sd from three biologically independent replicates. Statistically significant differences between different genotypes are denoted by distinct lowercase letters (*P* < 0.05, one-way ANOVA).

In Arabidopsis, CHR24 exhibits protein similarity with another Snf2-type remodeler, CHR8. Phylogenetic analysis reveals that they form a clade of the ERCC6 family along with mammalian ERCC6^CSB^ and yeast Rad26 (Fig. 3A). The predominant protein similarity is observed in their ATPase domains rather than in the remaining N- and C-termini (Supplementary Fig. S5). Notably, the expression level of *CHR8* is substantially lower than that of *CHR24* in vegetative organs, particularly in the shoot apex (Supplementary Fig. S6), which may explain why only CHR24 has been identified in our pulldown-MS using seedlings as materials.

We identified two T-DNA insertion mutants of *chr24* (*chr24-1*/SALK_152488 and *chr24-2*/SALK_050793) and one T-DNA insertion mutant of *chr8* (*chr8-1*/SAIL_381_A07), all of which are identified as loss-of-function mutants (Supplementary Fig. S7, A and B). The double mutant *chr8-1 chr24-1* (hereafter designated as Arabidopsis *ercc6*) was then generated. While no obvious growth phenotype was observed in any single mutant or the double mutant under the mock condition (Fig. 3, B and C; Supplementary Fig. S8), the *ercc6* double mutant displayed increased sensitivity to 5-FU treatment (Fig. 3, B and D). We used a biotin-coupled aldehyde reactive probe to detect the reactive deoxyribose that has lost bases (i.e. AP sites) (Fig. 3E). More AP sites were detected in genomic DNA extracted from the double mutant than in that from the single mutant and WT (Fig. 3F, Supplementary Fig. S8), suggestive of the impaired activity in AP elimination. All these defects of the *ercc6* mutant can be rescued when the entire genomic DNA of *CHR24* (including 2 kb of promoter and 1 kb of terminator) was reintroduced into the mutant (*ercc6/CHR24*) (Supplementary Fig. S9, A to C). We then examined the triple mutant *nap1;1-1 nap1;2-1 nap1;3-1* (hereafter designated as Arabidopsis *nap1-tri*) (Liu et al. 2009) (Supplementary Fig. S10). Consistent with the *ercc6* double mutant, the triple mutant *nap1-tri* also showed a wild-type growth phenotype as previously reported (Liu et al. 2009), but displayed hypersensitivity to 5-FU and higher AP levels in DNA (Fig. 3, B to F).

INOsitol auxotrophy 80 (INO80), another Snf2-type remodeler, has been implicated in plant growth and homologous recombination in DNA double-strand break repair (Zhang et al. 2015). NAP1-related protein 1 and 2 (NRP1/2), another H2A/H2B histone chaperone, possess similar protein domains with NAP1 but belong to different phylogeny groups (Zhu et al. 2006). We examined *ino80-5*, the null mutant of *INO80*, and the double mutant *nrp1-1 nrp2-1* in this study. Intriguingly, both mutants displayed normal resistance to 5-FU (Supplementary Fig. S9), highlighting the specificity of ERCC6 and NAP1 in the BER pathway triggered by 5-FU.

Notably, the *ercc6* double mutant and *nap1-tri* triple mutant, when crossed into the *ung* mutant (GK_440E07) (Cordoba-Canero et al. 2010), exhibited a comparable tolerance phenotype to 5-FU and basal AP level as *ung*. In contrast, when crossed into the *arp* mutant (SALK_021478) (Cordoba-Canero et al. 2011), both displayed a similar hypersensitivity to 5-FU as *arp* but an unusually high AP level compared to *arp* (Fig. 3, B to F). Our analysis reveals that the roles of CHR8/CHR24 and NAP1 in the BER pathway rely on UNG and ARP.

We crossed the double mutant *ercc6* with the triple mutant *nap1-tri* but failed to obtain a quintuple mutant after screening more than 1,000 F_3_ generation seedlings. We noticed that the chromosome location of *CHR8* (AT2G18760) is close to that of *NAP1;2* (AT2G19480) (Supplementary Fig. S11). Due to the unexpectedly strong linkage in these two adjacent genes, we eventually isolated the quadruple mutants *chr8-1 chr24-1 nap1;1-1 nap1;3-1* (*ercc6 nap1;1/3*) and *chr24-1 nap1;1-1 nap1;2-1 nap1;3-1* (*chr24 nap1-tri*) instead. The quadruple mutant *chr24 nap1-tri* but not *ercc6 nap1;1/3* showed obvious growth inhibition under normal growth condition (Fig. 3, B and C), suggesting that plant growth is sensitive to the functional loss of the predominant *CHR24* during the vegetative stage when *NAP1* genes are not expressed. Notably, both quadruple mutants displayed higher sensitivity to 5-FU (Fig. 3, B and D) and more AP lesions (Fig. 3F) than the double mutant *ercc6* and triple mutant *nap1-tri*. Collectively, our genetic and molecular analysis supports the notion that plant ERCC6 members and NAP1 proteins participate in a synergistic manner in the 5-FU-triggered and ARP-mediated BER pathway.

As previously reported, NAP1, as histone chaperone, can positively regulate the transcription of target genes through enrichment at these genes (Liu et al. 2009). Subsequently, we assessed the transcription level of *UNG*, *ARP*, *CHR8/CHR24*, and *NAP1* genes in 12 DAG seedlings subjected to either 5-FU or mock treatment. Notably, apart from a slight upregulation in *ercc6* double mutant for *NAP1;1* and *NAP1;2* transcripts, all the examined genes predominantly maintained wild-type expression levels (Supplementary Fig. S12), excluding the possibility that misregulated transcription of related genes during BER is primarily responsible for the impaired BER pathway in these mutants.

Furthermore, besides UNG-triggered uracil excision repair, ARP activity also participates in BER triggered by alkylating agent methyl methanesulfonate (MMS) (Akishev et al. 2016). The critical biological lesions induced by MMS are presumed to be the N-methylation base products, primarily as N7-methylguanine and N3-methyadenine DNA adducts, which frequently generate AP sites via enhanced hydrolysis of the N-glycosylic bond or DNA glycosylase-mediated base release (Wyatt and Pittman 2006). Intriguingly, both *ercc6* and *nap1-tri* mutants exhibited higher sensitivity toward MMS when compared to WT, and displayed similar hypersensitivity as *arp* when crossed into the *arp* background (Supplementary Fig. S13).

### Conservation of the ARP–ERCC6–NAP1 protein complex in yeast

**Figure 4.**
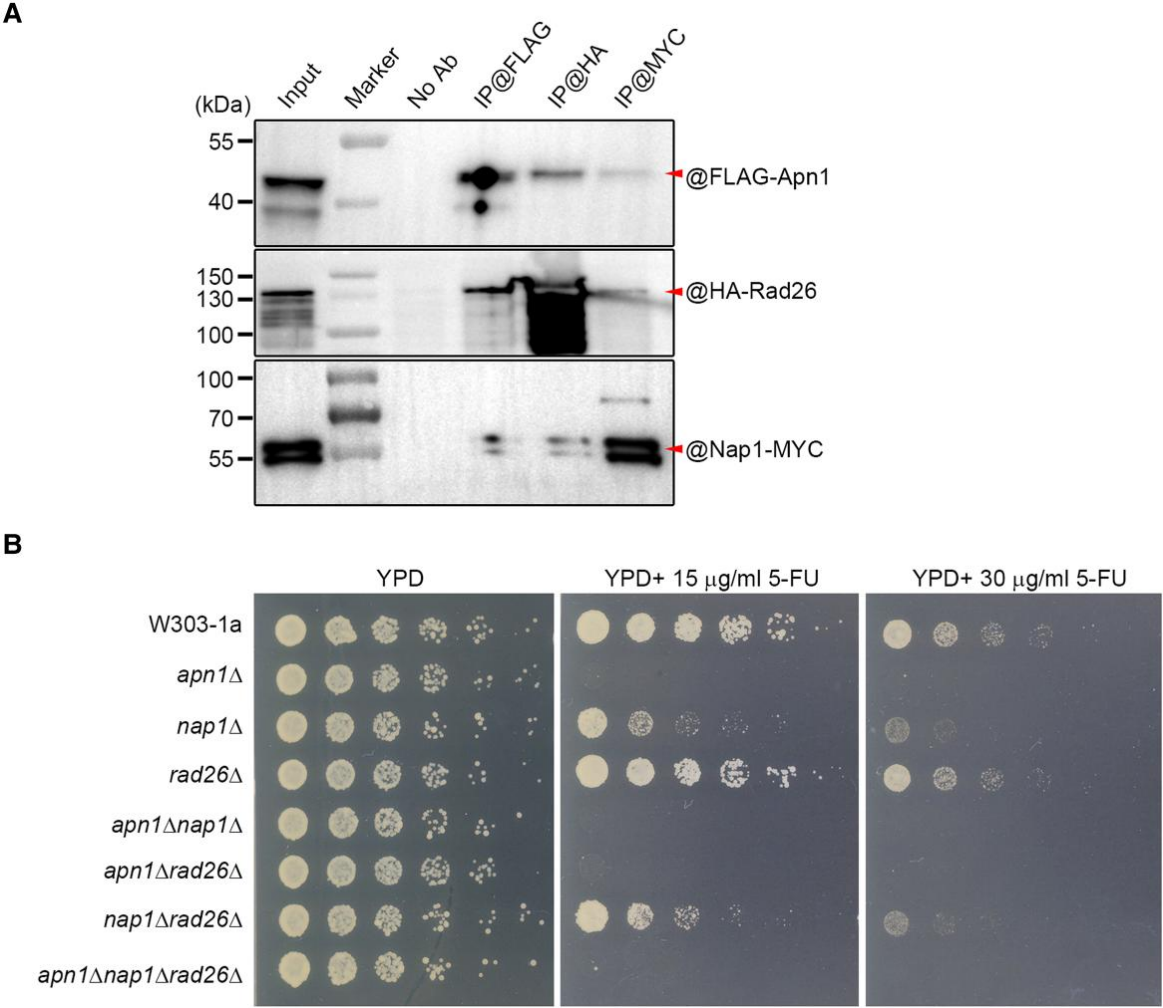
The conserved interaction of Apn1 with Nap1 and Rad26 in yeast. **A)** co-IP was performed to detect interactions between FLAG-Apn1, HA-Rad26, and Nap1-MYC. Total protein extracts from the yeast cells expressing tagged proteins were first immunoprecipitated with or without antibodies against FLAG, HA, and MYC, respectively, and the resulting fractions were then analyzed by immunoblotting. The arrows indicate the target protein bands, taking into consideration potential protein degradation or nonspecific bands observed in the immunoblot results. Notably, Nap1-MYC exhibits two closely sized bands in immunoblot analysis, and we speculate that small fragment may undergo splicing in the corresponding transcript within host cells. **B)** Spot assay of yeast strains (W303-1a as wild type) in the presence of 15 or 30 *μ*g/mL 5-FU.

The yeast genome also encodes ARP, NAP1, and ERCC6 homologue proteins (namely Apn1, Nap1, and Rad26, respectively). We co-expressed FLAG-Apn1, Nap1-MYC, and HA-Rad26 in yeast cells. In the co-IP experiment, we also found the mutual protein–protein interaction among these proteins in vivo (Fig. 4A), indicating that such mutual protein interaction is also conserved in yeast. Besides, we conducted pairwise co-IP validation of these three proteins, and our negative controls employed in these experiments substantiated the specificity of these interactions (Supplementary Fig. S14, A to C).

We then generated yeast strains depleted of these genes (i.e. *apn1*Δ, *nap1*Δ, and *rad26*Δ), as well as all three double mutants (i.e. *apn1*Δ*nap1*Δ, *apn1*Δ*rad26*Δ, and *nap1*Δ*rad26*Δ), and the triple mutant (*apn1*Δ*nap1*Δ*rad26*Δ) (Fig. 4B). Notably, all the yeast strains with *apn1*Δ exhibited hypersensitivity to 5-FU treatment, indicative of its dominant role in BER. As expected, the sensitivity of the *nap1*Δ strain to 5-FU is higher than that of the wild type (W303-1a), but lower than that of *apn1*Δ and *apn1*Δ*nap1*Δ, consistent with the potential involvement of Nap1 in Apn1-mediated BER. However, *rad26*Δ strain showed comparable sensitivity to 5-FU as W303-1a did, suggesting that it is dispensable in 5-FU-triggered BER. We further tested MMS-induced base damage stress. In this case, the *rad26*Δ strain displayed similar sensitivity to MMS as *apn1*Δ, while the *nap1*Δ strain showed a wild-type response (Supplementary Fig. S15). Moreover, *rad26*Δ and *apn1*Δ had a synergistic effect on MMS sensitivity, suggesting that Rad26 may not solely depend on Apn1 for its implication in the BER of methylated DNA bases. Taken together, Nap1 and Rad26 may function differently in Apn1-mediated BER repair depending on distinct types of base lesions despite similar mutual protein interactions as those observed in Arabidopsis.

## Discussion

The progression of BER is composed of multiple steps mediated by different enzymes, which coordinate the transfer of their repair intermediates (Fu et al. 2012; Endutkin et al. 2019). Prompt processing of AP site cleavage is crucial upon initiation of BER by monofunctional glycosylases, as these sites not only act as intermediates in BER but also contribute to the formation of DNA lesions with greater mutagenic and toxic severity, leading to genome instability (Thompson and Cortez 2020). Misregulation in BER has been broadly linked to human aging and various diseases (Wallace 2014). As a consequence, cells need to efficiently repair these AP sites to safeguard genome integrity, which are inevitably subject to regulation at the chromatin/nucleosome level.

Both the formation of DNA damage and its recognition by repair proteins in nucleosomes, the basic unit of DNA packaging in eukaryotic chromatin (Luger et al. 1997), occur differentially depending on rotational position, i.e. orientation in the DNA helix facing inwards or outwards, and strength of association between DNA and the octamer core (Caffrey and Delaney 2020). Reportedly, mammalian APE1 is generally inhibited by the presence of histones (Hinz and Czaja 2015), with further impairment of APE1 recognition of AP sites occurring in an orientation-specific manner (Hinz 2014, 2015). Although previous studies have detailed the effect of static reconstituted nucleosome structure on APE1 activity to each base, it should be noted that the relative position of DNA on natural nucleosomes in vivo will not remain constant. Transient or long-term exposure can be easily achieved via nucleosome remodeling, such as sliding. In this study, we demonstrate that histone chaperone NAP1 facilitates efficient sliding of nucleosomes by chromatin remodeler ERCC6, which exposes hindered endonuclease sites considerably improving cleavage efficiency in vitro. Based on our protein analysis, we speculate that ARP recruits ERCC6 and NAP1 through specific protein interactions to effectively circumvent inhibitory effects caused by the nucleosome structure on ARP activity. Consistent with this hypothesis, mutants in *ERCC6* and/or *NAP1* displayed enhanced *arp*-dependent sensitivity to base damage treatments.

A question is then raised as to why ARP requires chromatin-related factors to fulfill its activity while its upstream enzyme UNG does not. We propose several possible mechanisms. One is that the recognition of UNG to U is not dependent on any chromatin machinery, and that UNG can directly trap transiently exposed states arising from the rotational dynamics of DNA on histones. Ye et al. (2012) reported that the rotational position is not a strict factor to limit human UNG activity. Local dynamics of individual sites is likely to be involved in human UNG reactivity (Hinz et al. 2010). Moreover, human APE1 has been reported to stimulate the activity of human UDG in uracil base excision (Hinz et al. 2010). Therefore, despite the fact that Arabidopsis UNG does not physically interact with ERCC6/NAP1 (Supplementary Fig. S3), it is still possible that the sequential activities of UNG and ARP are tightly coupled so that ARP-recruited ERCC6/NAP1 activities are shared to facilitate the transient transition from uracil recognition to AP cleavage. Another possibility cannot be ruled out. Plant UNG may employ distinct chromatin factors instead of ERCC6/NAP1 to overcome the nucleosome barrier, which was not uncovered by our genetic experiments.

Increasing evidence has revealed the implication of mammalian ERCC6^CSB^ in diverse base lesion-triggered BER pathways through its protein interaction with distinct key enzymes in BER. Functionally, ERCC6^CSB^ interacts with and stimulates several bifunctional glycosylases, including OGG1 (Dianov et al. 1999; Tuo et al. 2002), NEIL1 (Muftuoglu et al. 2009) and NEIL2 (Aamann et al. 2014) which excises distinct base lesions. In addition, ERCC6^CSB^ also physically interacts with the unique, unstructured N-terminal domain of human APE1 and enhances its endonuclease activity using naked DNA templates (Wong et al. 2007). Intriguingly, the stimulatory effect of ERCC6^CSB^ on APE1 endonuclease activity is more profound in DNA bubble substrates resembling a DNA transcription intermediate rather than with the fully paired duplex, implying such stimulating mechanism primarily involves an alteration of the DNA double-helix conformation rather than the chromatin remodeling activity related to nucleosomes (Wong et al. 2007). Consistent with this, mammalian ERCC6^CSB^ and yeast putative ortholog Rad26 are well-known essential factors required for transcription-coupled repair and assist Pol II in overcoming downstream nucleosome barriers during transcription (Khobta et al. 2009; Xu et al. 2020). However, it should be noted that the BER pathway operates independent of Pol II-mediated transcription, since most nonbulky DNA lesions (e.g. uracil or AP site) can be bypassed by RNA polymerase II during transcriptional elongation without initiating the BER pathway (Kuraoka et al. 2003; Kathe et al. 2004; Charlet-Berguerand et al. 2006; Khobta et al. 2009). Instead, ERCC6^CSB^ has been found to act as an elongation factor to increase the efficiency of RNA pol II to read through oxidatively induced lesions, and therefore contribute to transcriptional mutagenesis (Charlet-Berguerand et al. 2006).

It should be emphasized here that plant ERCC6 protein has no effect on the endonuclease activity of ARP against naked DNA substrates in vitro (Supplementary Fig. S1B). In addition, the CHR24-ATPase domain requires a dynamic conformation capable of hydrolyzing ATP for its efficient interaction with ARP and NAP1 (Fig. 1F). Since ATPase activity of CHR24 is essential for nucleosome sliding (Fig. 2, D and F), we propose that ARP recruit catalytic remodelers to establish an optimal microenvironment of nucleosomal DNA for AP cleavage. Besides, although we did not validate the protein interaction between Arabidopsis ERCC6 and plant Pol II in this study, it is possible that a different mechanism other than BER could also be employed for plant ERCC6 recruitment and participation in transcription-coupled DNA repair, which remains to be elucidated by future analysis.

The N-terminal region of ERCC6^CSB^ exhibits an auto-repressive mechanism to negatively regulate both ATPase activity and stable association of ERCC6^CSB^ with chromatin. The relief of such autorepression is dependent on ATP hydrolysis, which is considered to drive a conformational change that favors its association with chromatin (Lake et al. 2010). The N-terminus of ERCC6^CSB^ is important to couple ATP hydrolysis to chromatin remodeling, and also crucial for its interaction with human NAP1L1 and NAP1L4, which is crucial for efficient transcription-coupled DNA repair (Cho et al. 2013). In this study, we demonstrate the conservation of ERCC6 and NAP1 interaction in plants and yeast. As a result, we propose that these two chromatin factors may form a functional module employed to remodel chromatin structure for efficient ARP/Apn1-mediated BER process. Notably, the poor conserved N-terminal region of human ERCC6^CSB^ compared to Arabidopsis ERCC6 and yeast Rad26 (Supplementary Fig. S5) suggests a distinct mechanism by which ERCC6 members dynamically regulate their quaternary structure with other associating partners via conformational change.

However, the flexibility of such a module cannot be ignored as several studies have revealed distinct combinations of chromatin remodelers and histone chaperones. For instance, NAP1 and REMODEL THE STRUCTURE OF CHROMATIN (RSC), a SWI/SNF family remodeler, has been reported to function in concert in nucleosomal 8-oxoG removal by using oligo-nucleosome substrates (Menoni et al. 2012). Moreover, upon oxidative stress, one human histone chaperone, FACILITATES CHROMATIN TRANSCRIPTION (FACT), is released from transcription-related protein complexes to associate with repair proteins and chromatin remodelers from the SWI/SNF family. FACT may collaborate with RSC to facilitate the excision of DNA lesions during the initial step of BER (Charles Richard et al. 2016). These findings raise a possibility that certain BER machineries may choose distinct modules from a toolbox to mobilize the nucleosome, the specificity of which is dependent on the mono- or bifunctional glycosylases and their following DNA repair processes in different species in vivo.

Indeed, although Rad26 and Nap1 form a similar protein complex, they appear to have inconsistent contributions to 5-FU and MMS-induced BER repair, while their plant homologs play a synergistic role in genotoxin resistance. Future biochemical and molecular analyses will be needed to elucidate how specific chromatin factors are recruited for distinct BER pathways at the chromatin level. This can improve our understanding of the co-evolution of epigenetic machineries and their regulatory mechanisms with chromatin architecture for genome maintenance across distinct organisms.

## Materials and methods

### Plant materials and growth conditions

Arabidopsis (*A. thaliana*) *arp-1* (SALK_021478) (Cordoba-Canero et al. 2011), *ung* (GK_440E07) (Cordoba-Canero et al. 2010), *chr8-1* (SAIL_381_A07), *chr24-1* (SALK_152488), and *chr24-2* (SALK_050793) were derived from the Columbia ecotype. The *nap1*-tri mutant crossed from *nap1;1-1* (SALK_013610), *nap1;2-1* (SAIL_84_B01), and *nap1;3-1* (SALK_131746) has been previously reported (Liu et al. 2009). In vitro plant culture was performed on agar-solidified Murashige and Skoog (MS) medium (M0255; Duchefa Biochemie, Haarlem, Netherlands) supplemented with 0.9% (w/v) sucrose. Plants were cultivated in a glasshouse at 22 °C under a 16-h:8-h light:dark condition. For BER-inducing treatment, seedlings were cultured in the presence of 150 *μ*m 5-FU (Cat No.HY-90006, Med Chem Express) or 125 *μ*L/L MMS (Cat No. 129925, MERCK). Arabidopsis mesophyll protoplast isolation and the transient gene expression were performed following a standard protocol (Yoo et al. 2007).

### Co-immunoprecipitation and pulldown assays

The co-IP experiment was performed as previously reported (Kang et al. 2019). One-tenth of the input and one-third of the immunoprecipitation (IP) fraction were separated on SDS–PAGE and detected by immunoblot using anti-FLAG (F3165; Sigma), anti-GFP (A-11122; Invitrogen), anti-MYC antibody (M20002L; Abmart) or anti-HA (ab9110; Abcam) at a dilution of 1:1,000. The pulldown experiment was performed as previously reported (Fan et al. 2022). Briefly, immobilized MBP–ARP and MBP (control) on Amylose Resin (E8021S, NEB) were mixed with plant extracts for 4 h at 4°C, followed by washing for three times, while GST-tagged proteins were immobilized on glutathione-sepharose 4B beads. The proteins remaining on the beads were resolved by SDS–PAGE, and examined in immunoblot.

### Mass spectrometric analyses

The pulldown proteins resolved in SDS–PAGE were silver stained, cut out from the gel, and subjected to trypsin digestion. The digested peptides were eluted on an analytical capillary column (75*μ*m × 20 *μ*m) packed with 2.4 *μ*m spherical C18 reversed-phase material. The eluted peptides were sprayed into an LTQ-Orbitrap Elite mass spectrometer (Thermo Fisher Scientific, Waltham, MA, USA) equipped with a nano-electrospray ionization ion source. Database searches were performed on the Mascot server (Matrix Science Ltd, London, UK) against the TAIR database. Pulldown-MS was performed in two biological replicates. MBP was used in pulldown-MS as a negative control. The screening criteria for those associated proteins was set at (1) protein score >50 and (2) no less than two unique peptides, in one replicate at least.

### ATPase assay

The ATPase activity was measured following the instructions of ATPase Activity Assay Kit (MAK113, Sigma) as previously reported (Kang et al. 2022b). The reaction was incubated at room temperature for 30 min, and terminated by addition of malachite green reagent. The colorimetric product was measured at 620 nm. Phosphate standards were performed in parallel.

### Nucleosome reconstitution and endonuclease accessibility assay

Reconstitution of nucleosomes were performed using human histone octamer (11010, Cyman) with a 225-bp DNA fragment including DpnII recognition site as previously reported (Du et al. 2020). The sequences of DNA templates and amplifying primers can be found in Supplementary Data Set 2. An AP site was introduced in 225-bp DNA template via PCR using AP-containing primer. Briefly, 225-bp DNA and histone octamer were dissolved in dialysis buffer (20 mm Tris–HCl, pH 7.4; 1 mm DTT; 1 mm EDTA, pH 8.0; 2 m KCl). Histone octamer and DNA were combined at a 0.9 m ratio and adjusted to a final DNA concentration of 6 *μ*m as previously reported (Nodelman et al. 2020). The reconstitution reaction mixture was placed in a Slide-A-Lyzer MINI Dialysis Device (Thermo, 69550). The dialysis was conducted in a beaker containing 1,000 mL of dialysis buffer and subjected to magnetic stirring at 4 °C. Subsequently, the KCl concentration was gradually reduced by slow pumping using a dialysis buffer with 0.125 m KCl over a period of 24 h. Following removal from the dialysis device, nucleosome reconstitution was evaluated via PAGE, employing the linear 225-bp DNA fragment as a control.

The endonuclease accessibility assay was performed as previously described (Kang et al. 2022b). The reactions were incubated at room temperature for the indicated time and then extracted by phenol:chloroform to remove proteins. For the DpnII site analysis, precipitated DNA was analyzed using a 6% PAGE gel, and visualized by Gel-Red (41003, Biotium). For the AP site analysis, precipitated DNA was resuspended in 10 *μ*L of 90% (v/v) formamide and subjected to heat treatment at 95 °C for 5 min. The resulting reaction products were separated using a 12% denaturing PAGE sequencing gel containing 7 m urea. Fluorescein-labeled DNA was visualized in an FLA-5100 imager.

### In vitro BER assay

Oligonucleotides used to prepare DNA substrates were synthesized by Sangon Biotech (https://www.sangon.com). Upper strand containing THF or cytosine (C) were labeled at the 5′ end with fluorescein. The upper strand was annealed to the lower strand by heating and slowly cooling to form a duplex DNA strand containing THG•G or C•G pair. In vitro BER assay was performed as previously reported (Cordoba-Canero et al. 2009). Briefly, repair reactions (50 *μ*L) contained 50 mm HEPES-KOH, pH 7.8, 70 mm KCl, 5 mm MgCl_2_, 1 mm DTT, 0.4 mm EDTA, 40 *μ*g BSA, 2% (v/v) glycerol, substrate DNA (40 nm), and indicated proteins. After incubation at 30 °C for 3 h, reactions were stopped by adding 20 mm EDTA, 0.6% (w/v) SDS and 0.5 mg/mL proteinase K, and the mixtures were incubated at 37 °C for 30 min. DNA was extracted with phenol:chloroform:isoamyl alcohol (25:24:1) and ethanol-precipitated at −20 °C in the presence of 0.3 mm NaCl and 16 *μ*g/mL glycogen. DNA were resuspended in 10 *μ*L of 90% (v/v) formamide and subjected to heat treatment at 95 °C for 5 min. The resulting reaction products were separated using a 12% denaturing PAGE sequencing gel containing 7 m urea. Fluorescein-labeled DNA was visualized in an FLA-5100 imager.

### Phylogenetic analysis

CHR24-related sequences were identified by BLAST searches mainly against the NCBI reference genome database. Multiple alignments of the amino acid sequences were performed using CLUSTALW version 2.0 (http://www.genome.jp/tools/clustalw/). The construction of the phylogenetic tree was performed using MEGA11 software with the neighbor-joining method, and phylogeny was tested with the bootstrap method. The sequence alignment and the tree file are provided as Supplementary Files 1 and 2.

### AP site counting assay

Genomic DNA was extracted following the manual (DP350, TIANGEN). AP site counting was performed following the protocol of the Aldehyde Reactive Probe (labeled with biotin) (#DK02, Dojindo, https://www.dojindo.cn/). The probe specifically reacts with aldehyde groups on the loop-opening of the AP site. These biotin-tagged AP sites can be quantified using an avidin–biotin assay followed by a colorimetric detection (650 nm) of peroxidase conjugated to the avidin.

### Preparation of yeast cells for co-IP

The triple mutant (*apn1*D *nap1*D *rad26*D) was transformed with plasmids expressing FLAG-Apn1, Nap1-MYC, and HA-Rad26. Transformants were grown at 30 °C in the synthetic complete medium lacking histidine, uracil, and tryptophan (SC-His-Ura-Trp) (Dunham et al. 2015) till an OD_600_ of ∼3. Cells were washed with H_2_O and then grown in SC-His-Ura-Trp medium containing 2% (w/v) raffinose instead of glucose for 8 h to relieve the glucose repression. Galactose was added into the medium to a final concentration of 2% (w/v) to induce the expression of HA-Rad26. Cells were collected after 10 h and resuspended in co-IP buffer (50 mm Tris–HCl, pH7.8, 100 mm NaCl, 5 mm MgCl_2_, 10% (v/v) glycerol, 0.05% (v/v) NP-40, 1 mm DTT, proteinase inhibitor cocktail (Roche)) in a concentration of 100 OD_600_/mL. Cells were mixed with acid-washed glass beads (G8772, Sigma–Aldrich) and processed by a bead-beater (FastPrep-24, MP, California, USA) at 6 m/s for 3 min. Lysate was centrifuged and the supernatant was subjected to the co-IP as described above.

### Spot assay of yeast cells

Cells were grown in the YPD plates (2% glucose, 2% polypeptone, 1% yeast extract, 2% agar, all v/v) for 1 d. Fresh cells were scraped from the plates and resuspended in the H_2_O to an OD_600_ of 1. The suspensions of cells were diluted by 5-fold four times. Dilutions were spotted on YPD plates containing 5-FU or MMS by a pinpad. Cells were grown for 2 d before imaging.

### Construction of plant and bacterial expression vectors

The coding sequences (CDS) of full-length CHR24, truncated CHR24-N, CHR24-ATPase, CHR24-ATPase^mut^, CHR24-C, and UNG were cloned into pGEX-6P-1 to express GST-tagged recombinant proteins. The CDS of ARP was cloned into pLM302 and transformed into Rosetta2 (DE3) to express MBP-tagged recombinant protein as previously reported (Lee et al. 2014). The CDS of ARP was cloned into pRTV-nMyc (He et al. 2018), and the CDS of CHR24 was cloned into pCAMBIA1306, and the CDS of NAP1;2 and RRM motif were cloned into pCAMBIA1301 (Fan et al. 2022) via recombination to express MYC-ARP, CHR24-FLAG, and YFP-NAP1; 2/YFP-RRM in planta, respectively. Notably, the expression of recombinant GST-CHR24-ATPase and -ATPase^mut^ proteins resulted in protein aggregation. Co-expression of chaperone plasmid pTf16 from Chaperone Plasmid Set (Takara, Code No. 3340) was required in the host bacteria cells.

### Construction of yeast strains and plasmids

Primers used in the constructions are listed in Supplementary Data Set 2. Yeast strains and plasmids are listed in Supplementary Data Set 3. To delete *NAP1*, the sequence targeting *NAP1* was inserted into a CRISPR vector, pRS425-Cas9-2xSapI. The resultant plasmid (LHZ969) was co-transformed into W303-1a with a donor sequence that ligated upstream and downstream sequences of *NAP1*. The resultant *nap1*Δ strain was named LHP1050. Similarly, *APN1* was deleted in W303-1a to obtain LHP1049. *RAD26* was deleted in W303-1a to obtain LHP1051. *NAP1* was deleted in LHP1051 to obtain LHP1054. *APN1* was deleted in LHP1050, LHP1051, and LHP1054, to obtain LHP1052, LHP1053, and LHP1055, respectively. To construct a plasmid expressing FLAG-Apn1 (LHZ932), the ORF of *APN1* with an N-terminal triple-*FLAG* tag was inserted behind a *TEF* promoter on a pRS426 vector. To construct a plasmid expressing HA-Rad26 (LHZ934), the ORF of *RAD26* with an N-terminal *HA* tag was inserted behind a *GAL1* promoter on a pRS423 vector. To construct a plasmid expressing Nap1-MYC (LHZ998), the ORF of *NAP1* with a C-terminal *MYC* tag was inserted behind a *TEF* promoter on a pRS424 vector. To construct a plasmid expressing MYC-GFP (LHZ1468), the ORF of *GFP* with a N-terminal *MYC* tag was inserted behind a *TEF* promoter on a pRS424 vector. To construct a plasmid expressing FLAG-mCherry (LHZ1469), the ORF of *mCherry* with an N-terminal triple-*FLAG* tag was inserted behind a *TEF* promoter on a pRS426 vector.

### Statistical analysis

Statistical analyses were conducted as specified in text and figure legends. Statistical data are provided in Supplementary Data Set S4.

### Accession numbers

Accession numbers of the genes used in this research are as follows: *ARP* (AT2G41460), *UNG* (AT3G18630), *CHR8* (AT2G18760), *CHR24* (AT5G63950), *INO80* (AT3G57300), *NAP1;1* (AT4G26110), *NAP1;2* (AT2G19480), *NAP1;3* (AT5G56950), *NRP1* (AT1G74560), and *NRP2* (AT1G18800).

## Supplementary Material

koae052_Supplementary_Data

## Data Availability

All data are available in the main text or Supplemental materials. The genetic materials supporting the findings of this study are available from the corresponding author upon reasonable request.

## Supplementary data

The following materials are available in the online version of this article.

**Supplementary Figure S1.** The in vitro BER assay.

**Supplementary Figure S2.** co-IP verification of protein–protein interactions among MYC-ARP, CHR24-FLAG, and YFP-NAP1;2.

**Supplementary Figure S3.** Pulldown assay using GST-UNG.

**Supplementary Figure S4.** GST-CHR24 specifically interacts with H3/H4 but not H2A/H2B in vitro.

**Supplementary Figure S5.** The protein similarity among CHR8/24, human ERCC6^CSB^ and yeast RAD26.

**Supplementary Figure S6.** The relative transcript levels of *CHR8* and *CHR24*.

**Supplementary Figure S7.** Characterization of T-DNA insertion mutant(s) of *CHR8* and *CHR24*.

**Supplementary Figure S8.** The *chr24-2* single mutant displayed wild-type sensitivity to 5-FU treatment.

**Supplementary Figure S9.** The mutants displayed wild-type sensitivity to 5-FU treatment.

**Supplementary Figure S10.** The updated nomenclature of each Arabidopsis *NAP1* gene and the corresponding mutant(s).

**Supplementary Figure S11.** The Arabidopsis chromosome location of two *ERCC6* genes and three *NAP1* genes.

**Supplementary Figure S12.** The sensitivity of *ercc6* and *nap1-tri* mutants to 5-FU is independent of the transcriptional change in *UNG* and *ARP*.

**Supplementary Figure S13.** The sensitivity of *ercc6* and *nap1-tri* mutants to MMS.

**Supplementary Figure S14.** co-IP verification of protein–protein interaction among Nap1-MYC, FLAG-Apn1, and HA-Rad26.

**Supplementary Figure S15.** The sensitivity of *apn1*Δ and *rad26*Δ strains to MMS.

**Supplementary Data Set 1.** The IP-MS results.

**Supplementary Data Set 2.** Primers used in this study.

**Supplementary Data Set 3.** Yeast strains and plasmids used in this study.

**Supplementary Data Set 4.** Statistical data.

**Supplementary File 1.** The sequence alignment (FASTA) for Fig. 3A.

**Supplementary File 2.** The tree file (nexus) for Fig. 3A.

## Dive Curated Terms

The following phenotypic, genotypic, and functional terms are of significance to the work described in this paper:

APE1 Gramene: AT5G38660
APE1 Araport: AT5G38660

OGG1 Gramene: AT1G21710
OGG1 Araport: AT1G21710

ARP Gramene: AT2G41460
ARP Araport: AT2G41460

NAP1;2 Gramene: AT2G19480
NAP1;2 Araport: AT2G19480

NAP1;1 Gramene: AT4G26110
NAP1;1 Araport: AT4G26110

NAP1;3 Gramene: AT5G56950
NAP1;3 Araport: AT5G56950

chr24 Gramene: AT5G63950
chr24 Araport: AT5G63950

chr8 Gramene: AT2G18760
chr8 Araport: AT2G18760

ape2 Gramene: At4g36050
ape2 Araport: At4g36050

## References

[koae052-B1] Aamann MD , Hvitby C, Popuri V, Muftuoglu M, Lemminger L, Skeby CK, Keijzers G, Ahn B, Bjoras M, Bohr VA, et al Cockayne syndrome group B protein stimulates NEIL2 DNA glycosylase activity. Mech Ageing Dev. 2014:135:1–14. 10.1016/j.mad.2013.12.008 24406253 PMC3954709

[koae052-B2] Akishev Z , Taipakova S, Joldybayeva B, Zutterling C, Smekenov I, Ishchenko AA, Zharkov DO, Bissenbaev AK, Saparbaev M. The major Arabidopsis thaliana apurinic/apyrimidinic endonuclease, ARP is involved in the plant nucleotide incision repair pathway. DNA Repair (Amst). 2016:48:30–42. 10.1016/j.dnarep.2016.10.009 27836324

[koae052-B3] Balliano AJ , Hayes JJ. Base excision repair in chromatin: insights from reconstituted systems. DNA Repair (Amst). 2015:36:77–85. 10.1016/j.dnarep.2015.09.009 26411876 PMC4688196

[koae052-B4] Bao Y , Shen X. INO80 subfamily of chromatin remodeling complexes. Mutat Res. 2007:618(1–2):18–29. 10.1016/j.mrfmmm.2006.10.006 17316710 PMC2699258

[koae052-B5] Beard BC , Wilson SH, Smerdon MJ. Suppressed catalytic activity of base excision repair enzymes on rotationally positioned uracil in nucleosomes. Proc Natl Acad Sci U S A. 2003:100(13):7465–7470. 10.1073/pnas.1330328100 12799467 PMC164609

[koae052-B6] Caffrey PJ , Delaney S. Chromatin and other obstacles to base excision repair: potential roles in carcinogenesis. Mutagenesis. 2020:35(1):39–50. 10.1093/mutage/gez029 31612219

[koae052-B7] Charles Richard JL , Shukla MS, Menoni H, Ouararhni K, Lone IN, Roulland Y, Papin C, Ben Simon E, Kundu T, Hamiche A, et al FACT assists base excision repair by boosting the remodeling activity of RSC. PLoS Genet. 2016:12(7):e1006221. 10.1371/journal.pgen.1006221 27467129 PMC4965029

[koae052-B8] Charlet-Berguerand N , Feuerhahn S, Kong SE, Ziserman H, Conaway JW, Conaway R, Egly JM. RNA polymerase II bypass of oxidative DNA damage is regulated by transcription elongation factors. EMBO J. 2006:25(23):5481–5491. 10.1038/sj.emboj.7601403 17110932 PMC1679758

[koae052-B9] Cho I , Tsai PF, Lake RJ, Basheer A, Fan HY. ATP-dependent chromatin remodeling by Cockayne syndrome protein B and NAP1-like histone chaperones is required for efficient transcription-coupled DNA repair. PLoS Genet. 2013:9(4):e1003407. 10.1371/journal.pgen.1003407 23637612 PMC3630089

[koae052-B10] Clapier CR , Cairns BR. The biology of chromatin remodeling complexes. Annu Rev Biochem. 2009:78(1):273–304. 10.1146/annurev.biochem.77.062706.153223 19355820

[koae052-B11] Clapier CR , Iwasa J, Cairns BR, Peterson CL. Mechanisms of action and regulation of ATP-dependent chromatin-remodelling complexes. Nat Rev Mol Cell Biol. 2017:18(7):407–422. 10.1038/nrm.2017.26 28512350 PMC8127953

[koae052-B12] Cordoba-Canero D , Dubois E, Ariza RR, Doutriaux MP, Roldan-Arjona T. Arabidopsis uracil DNA glycosylase (UNG) is required for base excision repair of uracil and increases plant sensitivity to 5-fluorouracil. J Biol Chem. 2010:285(10):7475–7483. 10.1074/jbc.M109.067173 20056608 PMC2844195

[koae052-B13] Cordoba-Canero D , Morales-Ruiz T, Roldan-Arjona T, Ariza RR. Single-nucleotide and long-patch base excision repair of DNA damage in plants. Plant J. 2009:60(4):716–728. 10.1111/j.1365-313X.2009.03994.x 19682284 PMC2954439

[koae052-B14] Cordoba-Canero D , Roldan-Arjona T, Ariza RR. Arabidopsis ARP endonuclease functions in a branched base excision DNA repair pathway completed by LIG1. Plant J. 2011:68(4):693–702. 10.1111/j.1365-313X.2011.04720.x 21781197

[koae052-B15] Dianov G , Bischoff C, Sunesen M, Bohr VA. Repair of 8-oxoguanine in DNA is deficient in Cockayne syndrome group B cells. Nucleic Acids Res. 1999:27(5):1365–1368. 10.1093/nar/27.5.1365 9973627 PMC148325

[koae052-B16] Du K , Luo Q, Yin L, Wu J, Liu Y, Gan J, Dong A, Shen WH. OsChz1 acts as a histone chaperone in modulating chromatin organization and genome function in rice. Nat Commun. 2020:11(1):5717. 10.1038/s41467-020-19586-z 33177521 PMC7658359

[koae052-B17] Duan M , Selvam K, Wyrick JJ, Mao P. Genome-wide role of Rad26 in promoting transcription-coupled nucleotide excision repair in yeast chromatin. Proc Natl Acad Sci U S A. 2020:117(31):18608–18616. 10.1073/pnas.2003868117 32690696 PMC7414089

[koae052-B18] Dunham MJ , Dunham MJ, Gartenberg MR, Brown GW. Methods in yeast genetics and genomics: a Cold Spring Harbor Laboratory course manual/Maitreya J. Dunham, University of Washington, Marc R. Gartenberg, Robert Wood Johnson Medical School, Rutgers, The State University of New Jersey, Grant W. Brown, University of Toronto. Cold Spring Harbor, New York: Cold Spring Harbor Laboratory Press; 2015.

[koae052-B19] Endutkin AV , Yudkina AV, Sidorenko VS, Zharkov DO. Transient protein-protein complexes in base excision repair. J Biomol Struct Dyn. 2019:37(17):4407–4418. 10.1080/07391102.2018.1553741 30488779

[koae052-B20] Fan T , Kang H, Wu D, Zhu X, Huang L, Wu J, Zhu Y. Arabidopsis gamma-H2A.X-INTERACTING PROTEIN participates in DNA damage response and safeguards chromatin stability. Nat Commun. 2022:13(1):7942. 10.1038/s41467-022-35715-2 36572675 PMC9792525

[koae052-B21] Fu D , Calvo JA, Samson LD. Balancing repair and tolerance of DNA damage caused by alkylating agents. Nat Rev Cancer. 2012:12(2):104–120. 10.1038/nrc3185 22237395 PMC3586545

[koae052-B22] Gao J , Zhu Y, Zhou W, Molinier J, Dong A, Shen WH. NAP1 family histone chaperones are required for somatic homologous recombination in Arabidopsis. Plant Cell. 2012:24(4):1437–1447. 10.1105/tpc.112.096792 22534127 PMC3407980

[koae052-B23] He F , Zhang F, Sun W, Ning Y, Wang GL. A versatile vector toolkit for functional analysis of rice genes. Rice (N Y). 2018:11(1):27. 10.1186/s12284-018-0220-7 29679176 PMC5910328

[koae052-B24] Hindi NN , Elsakrmy N, Ramotar D. The base excision repair process: comparison between higher and lower eukaryotes. Cell Mol Life Sci. 2021:78(24):7943–7965. 10.1007/s00018-021-03990-9 34734296 PMC11071731

[koae052-B25] Hinz JM . Impact of abasic site orientation within nucleosomes on human APE1 endonuclease activity. Mutat Res. 2014:766–767:19–24. 10.1016/j.mrfmmm.2014.05.008 PMC4112536 25847267

[koae052-B26] Hinz JM , Czaja W. Facilitation of base excision repair by chromatin remodeling. DNA Repair (Amst). 2015:36:91–97. 10.1016/j.dnarep.2015.09.011 26422134 PMC4688104

[koae052-B27] Hinz JM , Mao P, McNeill DR, Wilson DM III. Reduced nuclease activity of apurinic/apyrimidinic endonuclease (APE1) variants on nucleosomes: IDENTIFICATION OF ACCESS RESIDUES. J Biol Chem. 2015:290(34):21067–21075. 10.1074/jbc.M115.665547 26134573 PMC4543664

[koae052-B28] Hinz JM , Rodriguez Y, Smerdon MJ. Rotational dynamics of DNA on the nucleosome surface markedly impact accessibility to a DNA repair enzyme. Proc Natl Acad Sci U S A. 2010:107(10):4646–4651. 10.1073/pnas.0914443107 20176960 PMC2842065

[koae052-B29] Kang H , Fan T, Wu J, Zhu Y, Shen WH. Histone modification and chromatin remodeling in plant response to pathogens. Front Plant Sci. 2022a:13:986940. 10.3389/fpls.2022.986940 36262654 PMC9574397

[koae052-B30] Kang H , Liu Y, Fan T, Ma J, Wu D, Heitz T, Shen WH, Zhu Y. Arabidopsis CHROMATIN REMODELING 19 acts as a transcriptional repressor and contributes to plant pathogen resistance. Plant Cell. 2022b:34(3):1100–1116. 10.1093/plcell/koab318 34954802 PMC8894922

[koae052-B31] Kang H , Zhang C, An Z, Shen WH, Zhu Y. AtINO80 and AtARP5 physically interact and play common as well as distinct roles in regulating plant growth and development. New Phytol. 2019:223(1):336–353. 10.1111/nph.15780 30843208

[koae052-B32] Kathe SD , Shen GP, Wallace SS. Single-stranded breaks in DNA but not oxidative DNA base damages block transcriptional elongation by RNA polymerase II in HeLa cell nuclear extracts. J Biol Chem. 2004:279(18):18511–18520. 10.1074/jbc.M313598200 14978042

[koae052-B33] Khobta A , Kitsera N, Speckmann B, Epe B. 8-Oxoguanine DNA glycosylase (Ogg1) causes a transcriptional inactivation of damaged DNA in the absence of functional Cockayne syndrome B (Csb) protein. DNA Repair (Amst). 2009:8(3):309–317. 10.1016/j.dnarep.2008.11.006 19061977

[koae052-B34] Knizewski L , Ginalski K, Jerzmanowski A. Snf2 proteins in plants: gene silencing and beyond. Trends Plant Sci. 2008:13(10):557–565. 10.1016/j.tplants.2008.08.004 18786849

[koae052-B35] Krokan HE , Bjoras M. Base excision repair. Cold Spring Harb Perspect Biol. 2013:5(4):a012583–a012583. 10.1101/cshperspect.a012583 23545420 PMC3683898

[koae052-B36] Kuraoka I , Endou M, Yamaguchi Y, Wada T, Handa H, Tanaka K. Effects of endogenous DNA base lesions on transcription elongation by mammalian RNA polymerase II. Implications for transcription-coupled DNA repair and transcriptional mutagenesis. J Biol Chem. 2003:278(9):7294–7299. 10.1074/jbc.M208102200 12466278

[koae052-B37] Lake RJ , Geyko A, Hemashettar G, Zhao Y, Fan HY. UV-induced association of the CSB remodeling protein with chromatin requires ATP-dependent relief of N-terminal autorepression. Mol Cell. 2010:37(2):235–246. 10.1016/j.molcel.2009.10.027 20122405 PMC2818792

[koae052-B38] Lee J , Jang H, Shin H, Choi WL, Mok YG, Huh JH. AP endonucleases process 5-methylcytosine excision intermediates during active DNA demethylation in Arabidopsis. Nucleic Acids Res. 2014:42(18):11408–11418. 10.1093/nar/gku834 25228464 PMC4191409

[koae052-B39] Li J , Wang C, Liang W, Zhang J, Jiang CK, Liu Y, Ren Z, Ci D, Chang J, Han S, et al Functional importance and divergence of plant apurinic/apyrimidinic endonucleases in somatic and meiotic DNA repair. Plant Cell. 2023:35(6):2316–2331. 10.1093/plcell/koad056 36856605 PMC10226563

[koae052-B40] Licht CL , Stevnsner T, Bohr VA. Cockayne syndrome group B cellular and biochemical functions. Am J Hum Genet. 2003:73(6):1217–1239. 10.1086/380399 14639525 PMC1180389

[koae052-B41] Liu Z , Zhu Y, Gao J, Yu F, Dong A, Shen WH. Molecular and reverse genetic characterization of NUCLEOSOME ASSEMBLY PROTEIN1 (NAP1) genes unravels their function in transcription and nucleotide excision repair in Arabidopsis thaliana. Plant J. 2009:59(1):27–38. 10.1111/j.1365-313X.2009.03844.x 19228338

[koae052-B42] Luger K , Mäder AW, Richmond RK, Sargent DF, Richmond TJ. Crystal structure of the nucleosome core particle at 2.8 A resolution. Nature. 1997:389(6648):251–260. 10.1038/38444 9305837

[koae052-B43] Menoni H , Di Mascio P, Cadet J, Dimitrov S, Angelov D. Chromatin associated mechanisms in base excision repair—nucleosome remodeling and DNA transcription, two key players. Free Rad Biol Med. 2017:107:159–169. 10.1016/j.freeradbiomed.2016.12.026 28011149

[koae052-B44] Menoni H , Gasparutto D, Hamiche A, Cadet J, Dimitrov S, Bouvet P, Angelov D. ATP-dependent chromatin remodeling is required for base excision repair in conventional but not in variant H2A.Bbd nucleosomes. Mol Cell Biol. 2007:27(17):5949–5956. 10.1128/MCB.00376-07 17591702 PMC1952146

[koae052-B45] Menoni H , Shukla MS, Gerson V, Dimitrov S, Angelov D. Base excision repair of 8-oxoG in dinucleosomes. Nucleic Acids Res. 2012:40(2):692–700. 10.1093/nar/gkr761 21930508 PMC3258150

[koae052-B46] Muftuoglu M , de Souza-Pinto NC, Dogan A, Aamann M, Stevnsner T, Rybanska I, Kirkali G, Dizdaroglu M, Bohr VA. Cockayne syndrome group B protein stimulates repair of formamidopyrimidines by NEIL1 DNA glycosylase. J Biol Chem. 2009:284(14):9270–9279. 10.1074/jbc.M807006200 19179336 PMC2666579

[koae052-B47] Nakanishi S , Prasad R, Wilson SH, Smerdon M. Different structural states in oligonucleosomes are required for early versus late steps of base excision repair. Nucleic Acids Res. 2007:35(13):4313–4321. 10.1093/nar/gkm436 17576692 PMC1934998

[koae052-B48] Newman JC , Bailey AD, Weiner AM. Cockayne syndrome group B protein (CSB) plays a general role in chromatin maintenance and remodeling. Proc Natl Acad Sci U S A. 2006:103(25):9613–9618. 10.1073/pnas.0510909103 16772382 PMC1480455

[koae052-B49] Nilsen H , Lindahl T, Verreault A. DNA base excision repair of uracil residues in reconstituted nucleosome core particles. EMBO J. 2002:21(21):5943–5952. 10.1093/emboj/cdf581 12411511 PMC131078

[koae052-B50] Nisa MU , Huang Y, Benhamed M, Raynaud C. The plant DNA damage response: signaling pathways leading to growth inhibition and putative role in response to stress conditions. Front Plant Sci. 2019:10:653. 10.3389/fpls.2019.00653 31164899 PMC6534066

[koae052-B51] Nodelman IM , Patel A, Levendosky RF, Bowman GD. Reconstitution and purification of nucleosomes with recombinant histones and purified DNA. Curr Protoc Mol Biol. 2020:133(1):e130. 10.1002/cpmb.130 33305911 PMC7735289

[koae052-B52] Roldan-Arjona T , Ariza RR, Cordoba-Canero D. DNA base excision repair in plants: an unfolding story with familiar and novel characters. Front Plant Sci. 2019:10:1055. 10.3389/fpls.2019.01055 31543887 PMC6728418

[koae052-B53] Shaked H , Avivi-Ragolsky N, Levy AA. Involvement of the Arabidopsis SWI2/SNF2 chromatin remodeling gene family in DNA damage response and recombination. Genetics. 2006:173(2):985–994. 10.1534/genetics.105.051664 16547115 PMC1526515

[koae052-B54] Thompson PS , Cortez D. New insights into abasic site repair and tolerance. DNA Repair (Amst). 2020:90:102866. 10.1016/j.dnarep.2020.102866 32417669 PMC7299775

[koae052-B55] Troelstra C , van Gool A, de Wit J, Vermeulen W, Bootsma D, Hoeijmakers JH. ERCC6, a member of a subfamily of putative helicases, is involved in Cockayne’s syndrome and preferential repair of active genes. Cell. 1992:71(6):939–953. 10.1016/0092-8674(92)90390-X 1339317

[koae052-B56] Tuo J , Chen C, Zeng X, Christiansen M, Bohr VA. Functional crosstalk between hOgg1 and the helicase domain of Cockayne syndrome group B protein. DNA Repair (Amst). 2002:1(11):913–927. 10.1016/S1568-7864(02)00116-7 12531019

[koae052-B57] van den Boom V , Citterio E, Hoogstraten D, Zotter A, Egly JM, van Cappellen WA, Hoeijmakers JH, Houtsmuller AB, Vermeulen W. DNA damage stabilizes interaction of CSB with the transcription elongation machinery. J Cell Biol. 2004:166(1):27–36. 10.1083/jcb.200401056 15226310 PMC2172148

[koae052-B58] van der Horst GT , van Steeg H, Berg RJ, van Gool AJ, de Wit J, Weeda G, Morreau H, Beems RB, van Kreijl CF, de Gruijl FR, et al Defective transcription-coupled repair in Cockayne syndrome B mice is associated with skin cancer predisposition. Cell. 1997:89(3):425–435. 10.1016/S0092-8674(00)80223-8 9150142

[koae052-B59] Vertessy BG , Toth J. Keeping uracil out of DNA: physiological role, structure and catalytic mechanism of dUTPases. Acc Chem Res. 2009:42(1):97–106. 10.1021/ar800114w 18837522 PMC2732909

[koae052-B60] Wallace SS . Base excision repair: a critical player in many games. DNA Repair (Amst). 2014:19:14–26. 10.1016/j.dnarep.2014.03.030 24780558 PMC4100245

[koae052-B61] Wong HK , Muftuoglu M, Beck G, Imam SZ, Bohr VA, Wilson DM III. Cockayne syndrome B protein stimulates apurinic endonuclease 1 activity and protects against agents that introduce base excision repair intermediates. Nucleic Acids Res. 2007:35(12):4103–4113. 10.1093/nar/gkm404 17567611 PMC1919475

[koae052-B62] Wyatt MD , Pittman DL. Methylating agents and DNA repair responses: methylated bases and sources of strand breaks. Chem Res Toxicol. 2006:19(12):1580–1594. 10.1021/tx060164e 17173371 PMC2542901

[koae052-B63] Wyatt MD , Wilson DM III. Participation of DNA repair in the response to 5-fluorouracil. Cell Mol Life Sci. 2009:66(5):788–799. 10.1007/s00018-008-8557-5 18979208 PMC2649968

[koae052-B64] Xu J , Wang W, Xu L, Chen JY, Chong J, Oh J, Leschziner AE, Fu XD, Wang D. Cockayne syndrome B protein acts as an ATP-dependent processivity factor that helps RNA polymerase II overcome nucleosome barriers. Proc Natl Acad Sci U S A. 2020:117(41):25486–25493. 10.1073/pnas.2013379117 32989164 PMC7568279

[koae052-B65] Ye Y , Stahley MR, Xu J, Friedman JI, Sun Y, McKnight JN, Gray JJ, Bowman GD, Stivers JT. Enzymatic excision of uracil residues in nucleosomes depends on the local DNA structure and dynamics. Biochemistry. 2012:51(30):6028–6038. 10.1021/bi3006412 22784353 PMC3448002

[koae052-B66] Yoo SD , Cho YH, Sheen J. Arabidopsis mesophyll protoplasts: a versatile cell system for transient gene expression analysis. Nat Protoc. 2007:2(7):1565–1572. 10.1038/nprot.2007.199 17585298

[koae052-B67] Zhang C , Cao L, Rong L, An Z, Zhou W, Ma J, Shen WH, Zhu Y, Dong A. The chromatin-remodeling factor AtINO80 plays crucial roles in genome stability maintenance and in plant development. Plant J. 2015:82(4):655–668. 10.1111/tpj.12840 25832737

[koae052-B68] Zhou W , Zhu Y, Dong A, Shen WH. Histone H2A/H2B chaperones: from molecules to chromatin-based functions in plant growth and development. Plant J. 2015:83(1):78–95. 10.1111/tpj.12830 25781491

[koae052-B69] Zhu Y , Dong A, Meyer D, Pichon O, Renou JP, Cao K, Shen WH. (2006). Arabidopsis NRP1 and NRP2 encode histone chaperones and are required for maintaining postembryonic root growth. Plant Cell 18(11), 2879–2892. doi:10.1105/tpc.106.046490 17122067 PMC1693930

