# Supplementary material for: The chromatin remodeler ERCC6 and the histone chaperone NAP1 are involved in apurinic/apyrimidinic endonuclease-mediated DNA repair
Source: Plant Cell. 2024 Feb 17;36(6):2238–52. doi: 10.1093/plcell/koae052 (PMC11132878; doi:10.1093/plcell/koae052)
Supplement: koae052_Supplementary_Data [file koae052_supplementary_data.zip › TPC2023RA01027R2_Supplemental Figures 115V1.pdf]

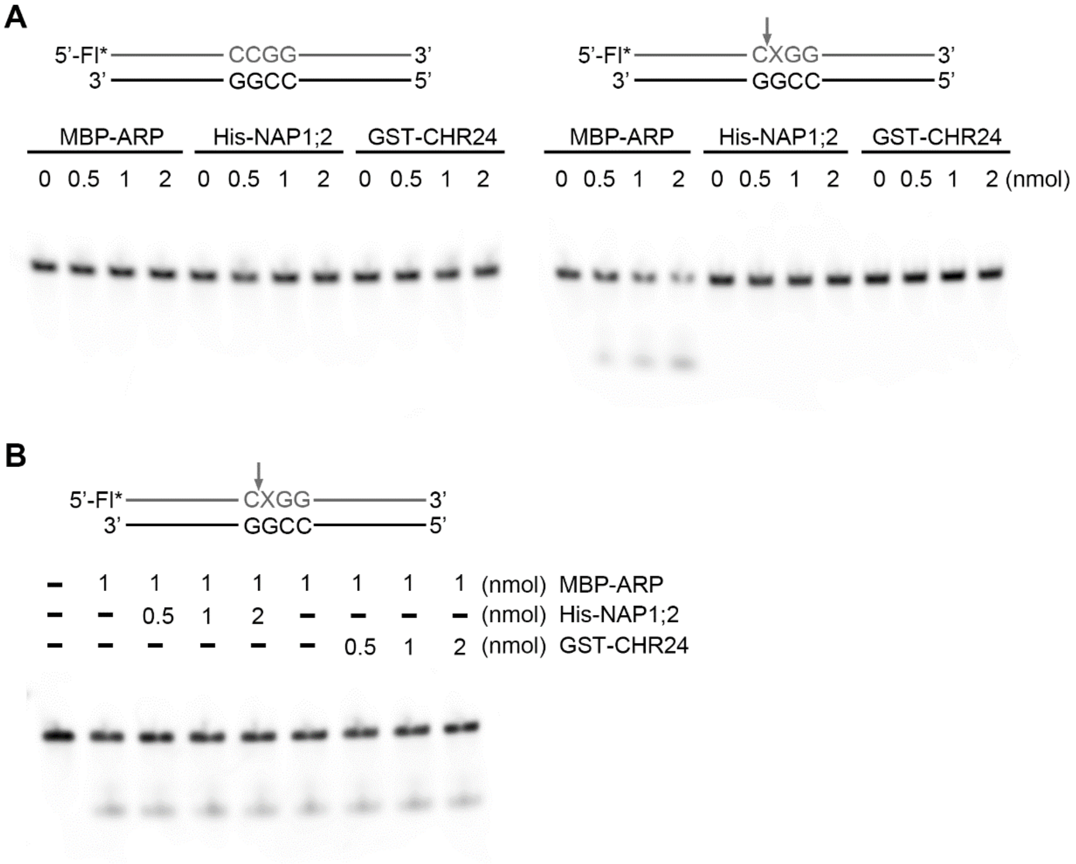

Supplemental Figure S1. The *in vitro* BER assay. (Supports Figure 1 and 2)

(A) MBP-ARP exhibits AP endonuclease activity specific to the X:G (tetrahydrofuran against G) but not C:G pair, while His-NAP1;2 or GST-CHR24 showed no endonuclease activity.

(B) Addition of His-NAP1;2 or GST-CHR24 cannot stimulate the endonuclease activity of ARP in the *in vitro* BER assay.

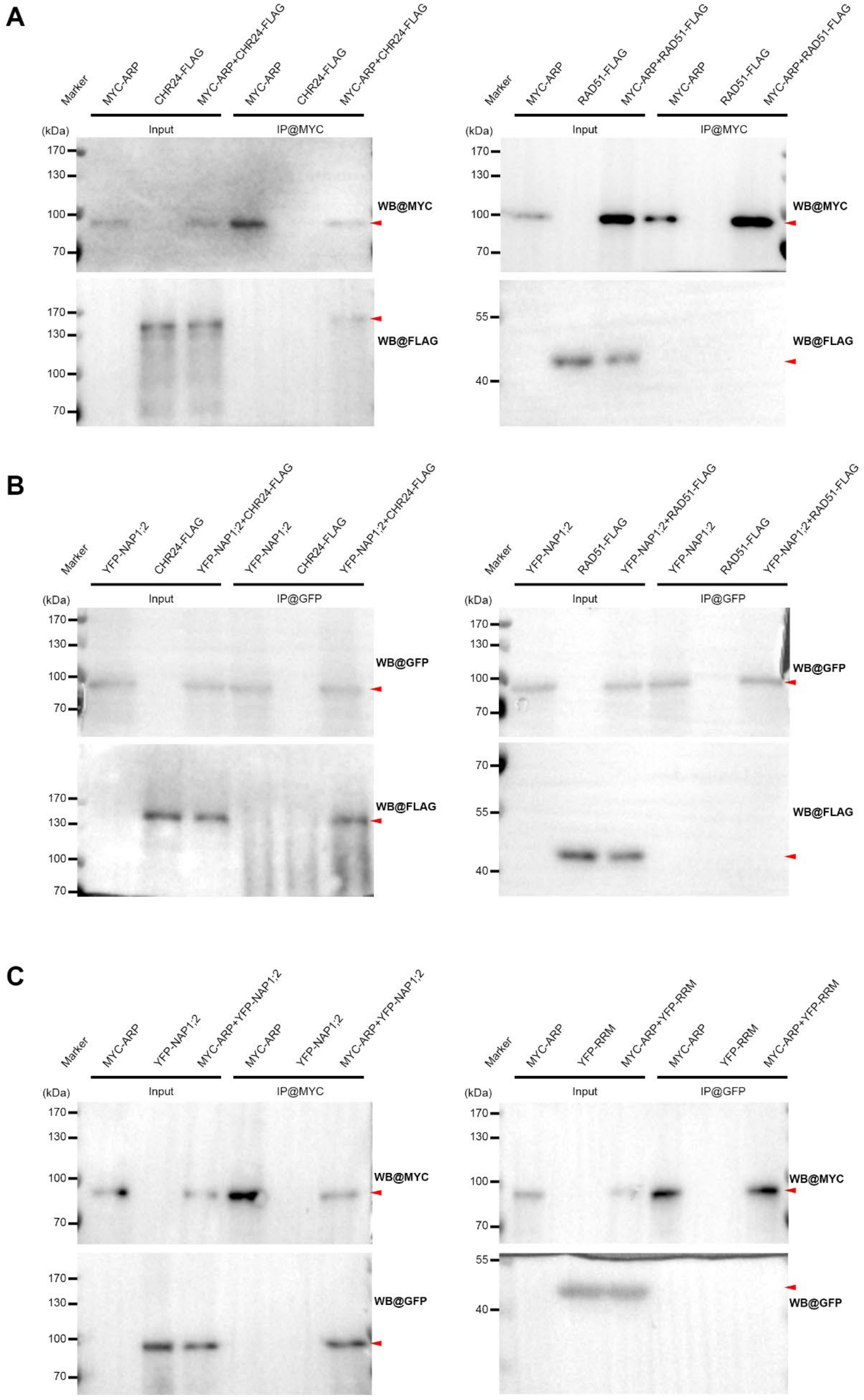
A
Marker
MYC-ARP
CHR24-FLAG
MYC-ARP+CHR24-FLAG
MYC-ARP
CHR24-FLAG
MYC-ARP+CHR24-FLAG
Input
IP@MYC
(kDa)
170
130
100
70
WB@MYC
WB@FLAG
Marker
MYC-ARP
RAD51-FLAG
MYC-ARP+RAD51-FLAG
MYC-ARP
RAD51-FLAG
MYC-ARP+RAD51-FLAG
Input
IP@MYC
55
40
B
YFP-NAP1;2
CHR24-FLAG
YFP-NAP1;2+CHR24-FLAG
IP@GFP
WB@GFP
WB@FLAG
RAD51-FLAG
YFP-NAP1;2+RAD51-FLAG
C
MYC-ARP+YFP-NAP1;2
YFP-RRM
MYC-ARP+YFP-RRM

Supplemental Figure S2. co-IP verification of protein-protein interactions among MYC-ARP, CHR24-FLAG and YFP-NAP1;2. (Supports Figure 1)

(A) Co-immunoprecipitation was performed to detect the interactions between MYC-ARP and CHR24-FLAG *in planta*. Total protein extracts from the mesophyll protoplasts expressing tagged proteins were first immunoprecipitated with antibodies against MYC and FLAG, respectively, followed by immunoblot analysis of resulting fractions. RAD51-FLAG (**Fan et al., 2022**) was used as the negative control of CHR24-FLAG. The red arrowheads indicate the target protein bands, taking into consideration potential protein degradation or non-specific bands observed in the immunoblot results.

(B) The interactions between YFP-NAP1;2 and CHR24-FLAG *in planta*. RAD51-FLAG (**Fan et al., 2022**) was used as the negative control of CHR24-FLAG.

(C) The interactions between MYC-ARP and YFP-NAP1;2 *in planta*. The overexpression of *rFCA* RRM (RNA recognition motifs) can regulate cell size in rice (**Hong et al., 2007**). Functional RRM was cloned from Arabidopsis *FCA* gene (At4g16280) and expressed as a YFP-tagged protein as the negative control of YFP-NAP1;2.

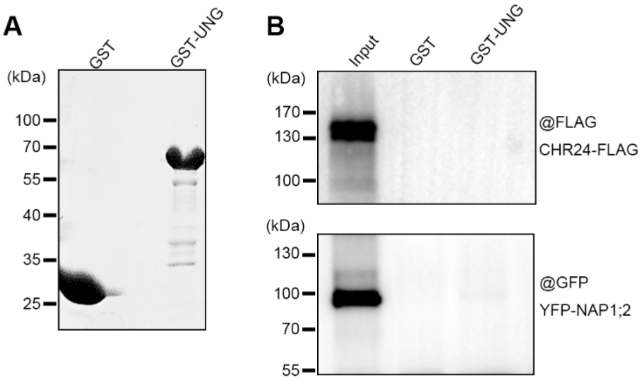

Supplemental Figure S3. Pulldown assay using GST-UNG. (Supports Figure 1)

(A) Purified GST and GST-tagged UNG (GST-UNG) proteins in Coomassie brilliant blue-stained SDS-PAGE gel.

(B) Immobilized GST-UNG and GST (control) were incubated with protein extracts from mesophyll protoplasts expressing YFP-NAP1;2 and CHR24-FLAG.

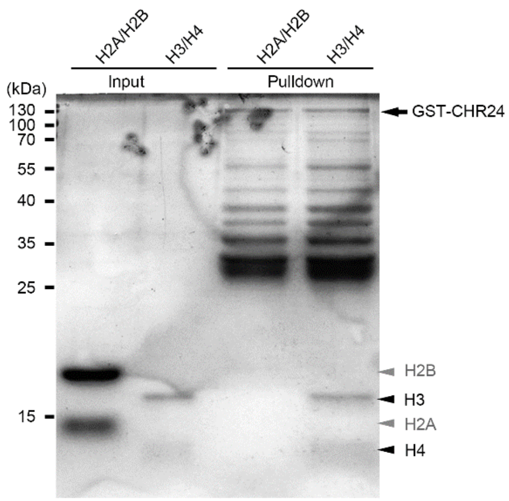

Supplemental Figure S4. GST-CHR24 specifically interacts with H3/H4 but not H2A/H2B *in vitro*. (Supports Figure 2)
Immobilized GST-CHR24 was incubated with recombinant plant histones H2A, H2B, H3 and H4, which were expressed and purified from BL21 (DE3) as previously reported (**Luo et al., 2020**). Notably, although the H2A/H2B dimer was overloaded, only H3/H4 histones were retrieved by GST-CHR24.

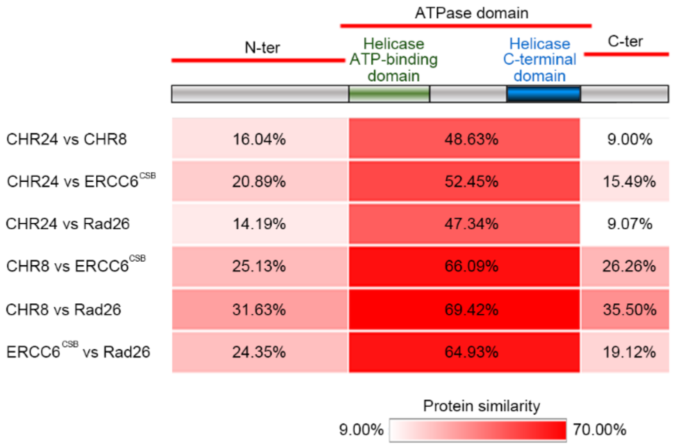

Supplemental Figure S5. The protein similarity among CHR8/24, human ERCC6^{CSB} and yeast RAD26. (Supports Figure 3)
The intact ATPase domains were analyzed using the online tools Pfam (http://pfam.sanger.ac.uk/) and SMART (http://smart.embl-heidelberg.de/). The similarity of N-termini (N-ter), ATPase domains and C-termini (C-ter) among these related remodelers were analyzed using the online tool Sequence Manipulation Suite (SMS2, http://www.detaibio.com/sms2/mirror.html)

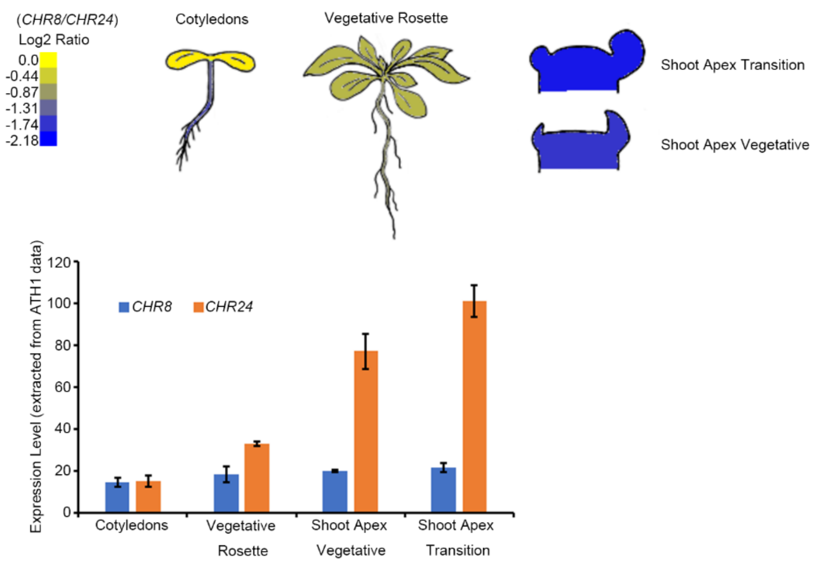

Supplemental Figure S6. The relative transcript levels of *CHR8* and *CHR24*. (Supports Figure 3)

The relative transcript levels of *CHR8* to *CHR24* were obtained from the Arabidopsis eFP Browser, a publicly available database (http://bar.utoronto.ca/efp/cgi-bin/efpWeb.cgi). The expression levels and the standard deviation of *CHR8* and *CHR24* are directly calculated and provided by eFP Browser based on at least three transcriptome replicates.

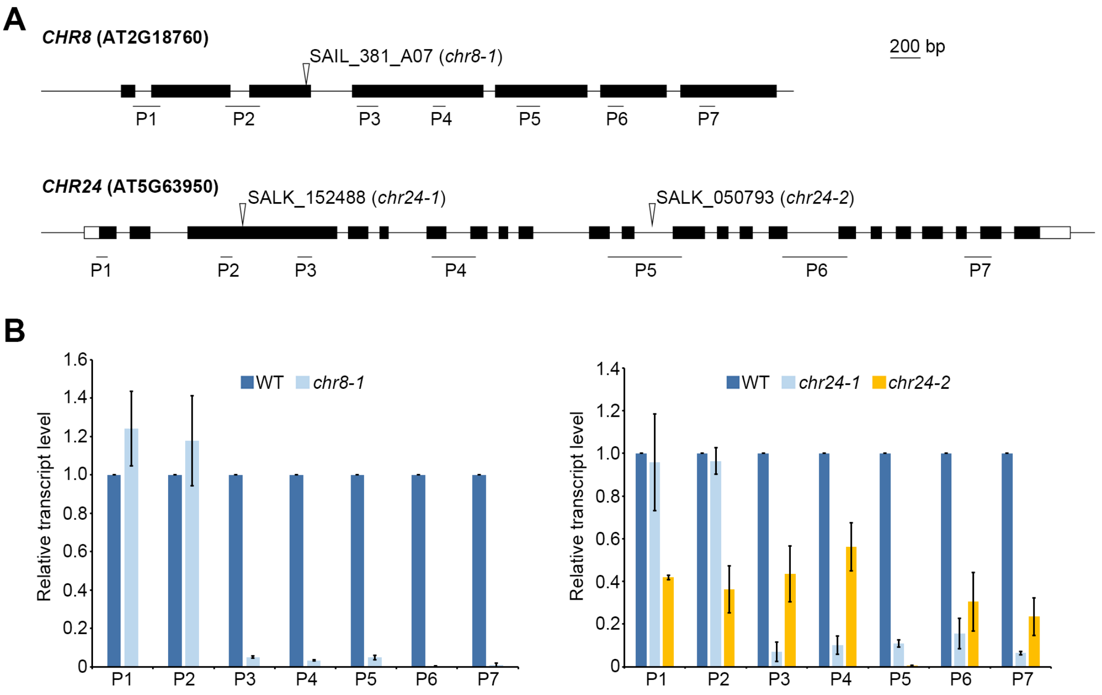

Supplemental Figure S7. Characterization of T-DNA insertion mutant(s) of *CHR8* and *CHR24*. (Supports Figure 3)

(A) Schematic representation of the *CHR8* and *CHR24* genes, and the T-DNA insertions in *chr8-1* (SAIL_381_A07), *chr24-1* (SALK_152488) and *chr24-2* (SALK_050793). The gene structure is indicated by black and white boxes representing coding sequences and untranslated regions, respectively. Number-labeled bars (P1–P7) correspond to regions amplified by corresponding primer pairs for transcript detection in (**B**).

(B) RT-qPCR analysis of *CHR8/CHR24* transcripts in 12-day-old seedlings. *ACT2* was used as the reference gene. Relative expression levels of *CHR8/CHR24* transcripts were further normalized to those in the WT (set as 1). Mean values are shown with error bars from three independent experiments.

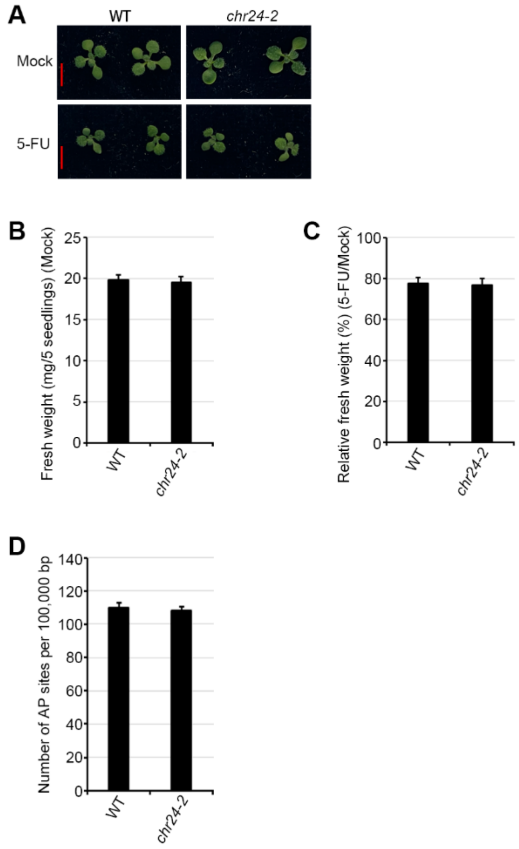

Supplemental Figure S8. The *chr24-2* single mutant displayed wild-type sensitivity to 5-FU treatment. (Supports Figure 3)

(A) Images of 12-day-old plants grown on medium with or without 5-FU. Bar=10 mm.

(B) The fresh weight of 5 seedlings grown on medium without 5-FU (Mock treatment) as one biological replicate. Mean values are presented along with error bars indicating ±SD from 10 biologically independent replicates. The comparison between the WT and mutant did not yield any statistically significant differences ($P > 0.05$).

(C) Comparison of the plant sensitivities to 5-FU by normalizing the fresh weights of plants grown with 5-FU to those of plants grown with mock treatment (as one biological replicate). Mean values are shown together with error bars indicating ±SD from 10 biologically independent replicates.

(D) Comparison of the AP level in the genomic DNA extracted from plants in (**A**). Mean values are shown together with error bars indicating ±SD from 3 biologically independent replicates.

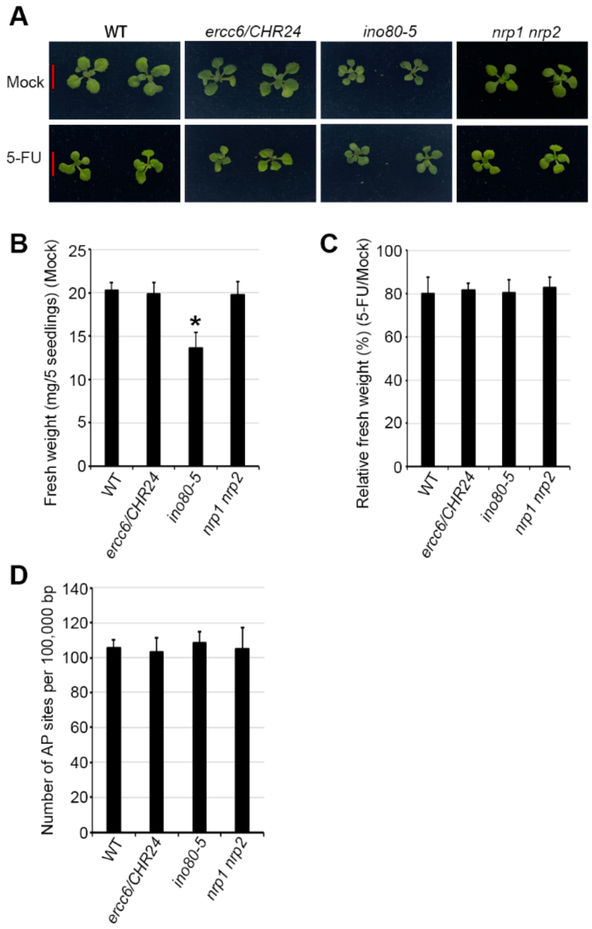

Supplemental Figure S9. The mutants displayed wild-type sensitivity to 5-FU treatment. (Supports Figure 3)

(A) Images of 12-day-old plants grown on medium with or without 5-FU. The *ercc6* mutant complemented with the genomic DNA of *CHR24* was designated as *ercc6/CHR24*. Bar=10 mm.

(B) The wet weight of 5 seedlings grown on medium without 5-FU (Mock treatment) as one biological replicate. Mean values are presented along with error bars indicating ±SD from 10 biologically independent replicates. The asterisk indicates a significant difference between the WT and mutant ($P < 0.05$).

(C) Comparison of the plant sensitivities to 5-FU by normalizing the wet weights of plants grown with 5-FU to those of plants grown with mock treatment (as one biological replicate). Mean values are shown together with error bars indicating ±SD from 10 biologically independent replicates.

(D) Comparison of the AP level in the genomic DNA extracted from plants in (**A**). Mean values are shown together with error bars indicating ±SD from 3 biologically independent replicates.

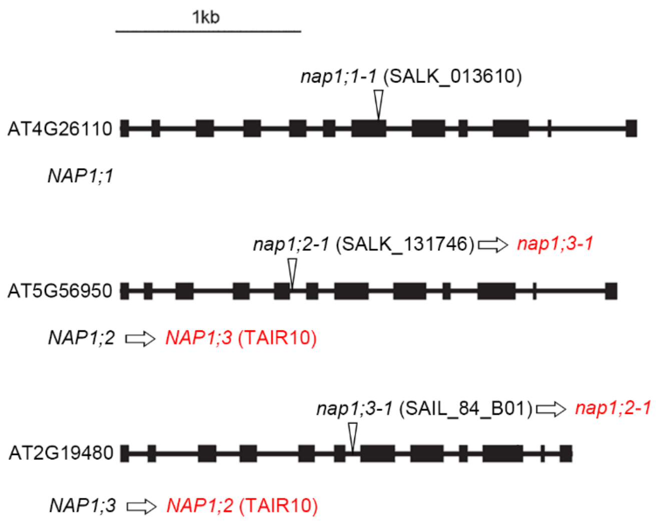

Supplemental Figure S10. The updated nomenclature of each Arabidopsis *NAP1* gene and the corresponding mutant(s). (Supports Figure 3)
Notably, the original nomenclature of these three *NAP1* genes (black) (**Liu et al., 2009**) became confusing after the release of Araport11 (TAIR10). In this study, we updated the information of these genes and corresponding mutants (red).

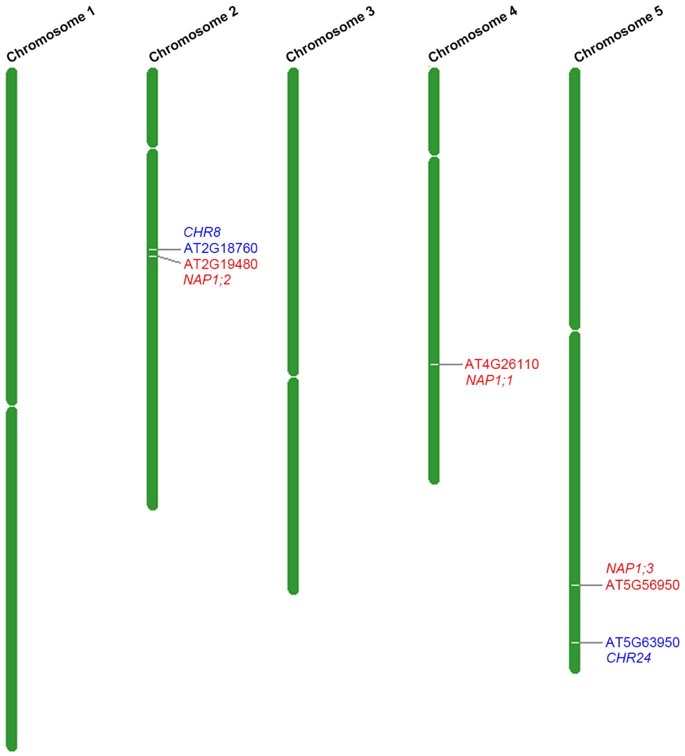

Supplemental Figure S11. The Arabidopsis chromosome location of two *ERCC6* genes and three *NAP1* genes. (Supports Figure 3)

Note that *CHR8* (AT2G18760) exhibits strong genetic linkage with *NAP1;2* (AT2G19480) due to their close proximity. Additionally, *CHR24* (AT5G63950) and *NAP1;3* (AT5G56950) are located on the same arm of chromosome 5, resulting in moderate genetic linkage.

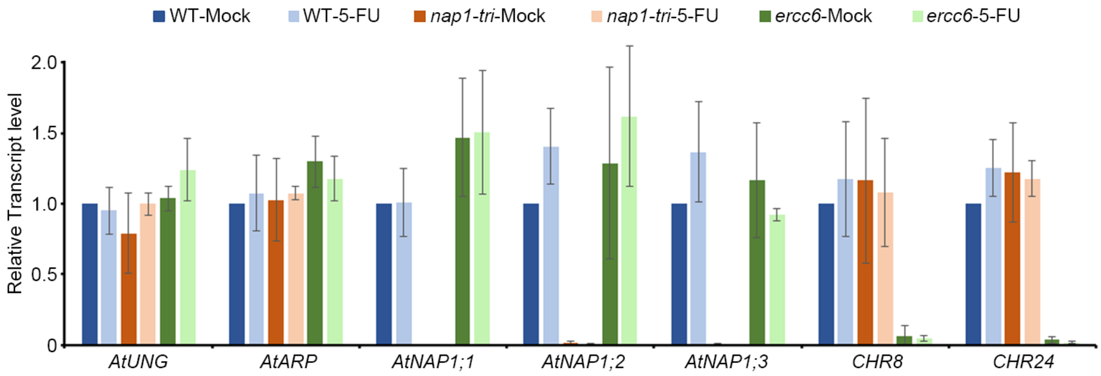

Supplemental Figure S12. The sensitivity of *ercc6* and *nap1-tri* mutants to 5-FU is independent of the transcriptional change in *UNG* and *ARP*. (Supports Figure 3)

The relative transcript levels of the selected genes were determined using *ACT2* as the reference gene. Mean values are shown together with error bars indicating ±SD from three independent biological replicates.

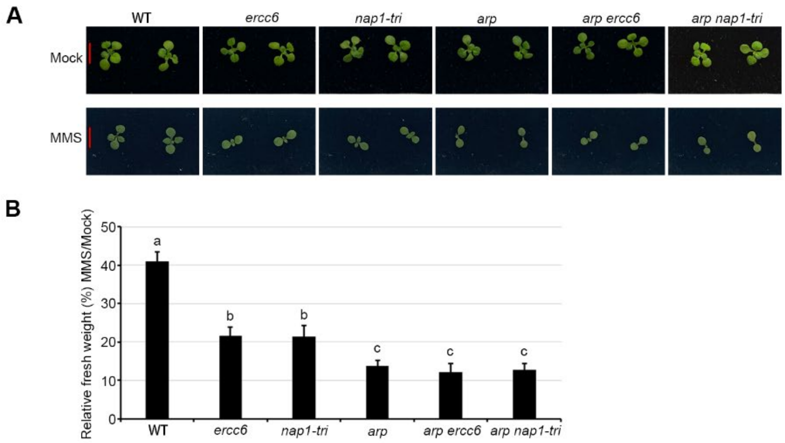

Supplemental Figure S13. The sensitivity of *ercc6* and *nap1-tri* mutants to methyl methanesulfonate. (Supports Figure 3)

(A) Images of 12-day-old plants grown on the medium with or without 125 μL/L methyl methanesulfonate (MMS). Bar=10 mm.

(B) Comparison of the plant sensitivities to MMS by the normalization of the fresh weights of plants grown with MMS to those of plants grown with mock treatment. Mean values are shown together with error bars indicating ±SD from 10 biologically independent replicates. Statistically significant differences between different genotypes are denoted by distinct lowercase letters ($P < 0.05$, one-way ANOVA).

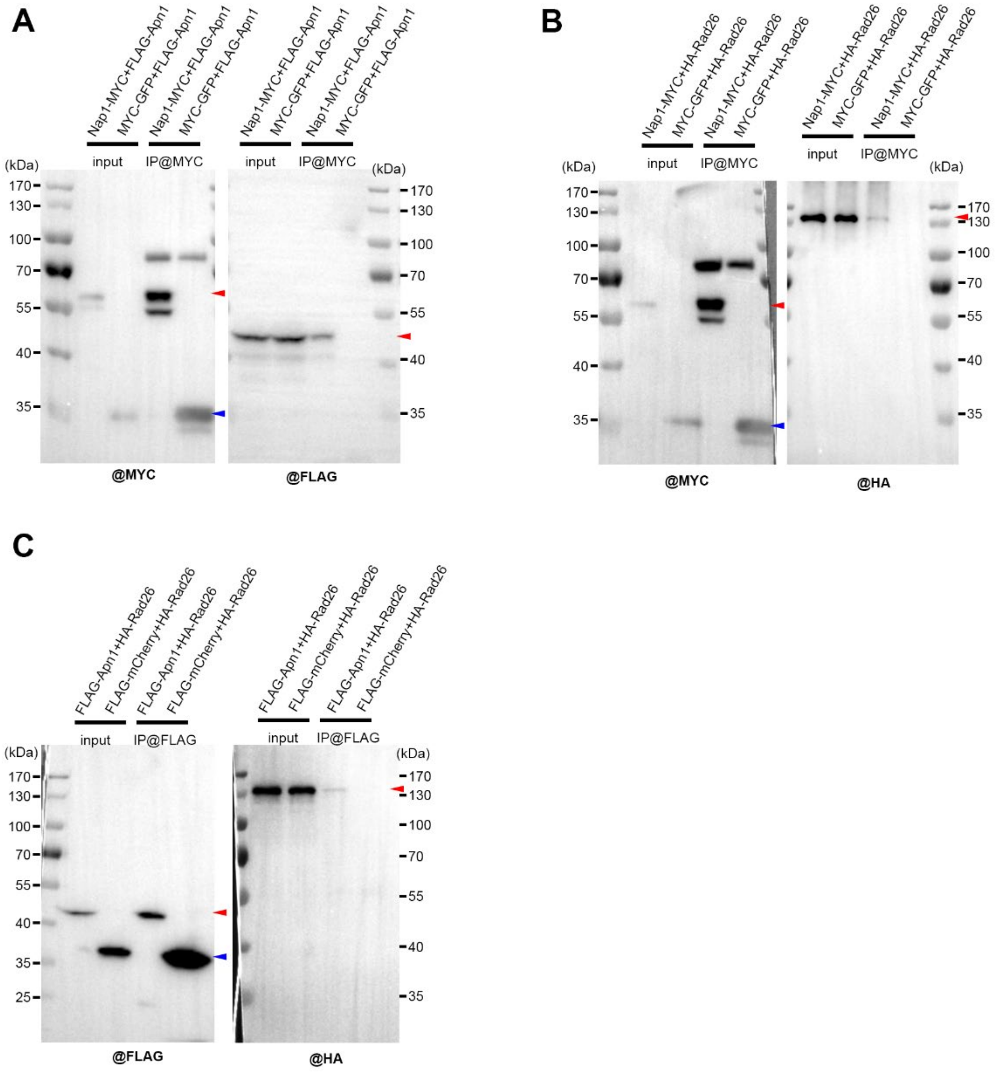

Supplemental Figure S14. co-IP verification of protein-protein interaction among Nap1-MYC, FLAG-Apn1 and HA-Rad26. (Supports Figure 4)

(A) Co-immunoprecipitation was performed to detect the interactions between Nap1-MYC and FLAG-Apn1 *in vivo*. Total protein extracts from the yeast cells expressing tagged proteins were first immunoprecipitated with antibodies against MYC and FLAG, respectively, followed by immunoblot analysis of resulting fractions. MYC-GFP was constructed and used as the negative control of Nap1-MYC. The red and blue arrowheads indicate the target and control protein bands, respectively, taking into consideration potential protein degradation or non-specific bands observed in the immunoblot results.

(B) The interactions between Nap1-MYC and HA-Rad26 *in vivo*. MYC-GFP was used as the negative control of CHR24-FLAG.

(C) The interactions between FLAG-Apn1 and HA-Rad26 *in vivo*. FLAG-mCherry was constructed and used as the negative control of FLAG-Apn1.

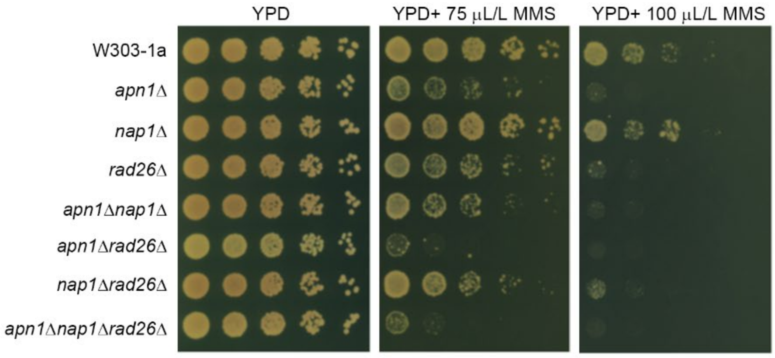

Supplemental Figure S15. The sensitivity of *apn1Δ* and *rad26Δ* strains to methyl methanesulfonate. (Supports Figure 4)
Spot assay of yeast strains (W303-1a as wild type) in the presence of 75 and 100 µL/L methyl methanesulfonate (MMS).

Supplemental references

Fan, T., Kang, H., Wu, D., Zhu, X., Huang, L., Wu, J., and Zhu, Y. (2022). Arabidopsis gamma-H2A.X-INTERACTING PROTEIN participates in DNA damage response and safeguards chromatin stability. Nature communications **13,** 7942.

Hong, F., Attia, K., Wei, C., Li, K., He, G., Su, W., Zhang, Q., Qian, X., and Yang, J. (2007). Overexpression of the rFCA RNA recognition motif affects morphologies modifications in rice (Oryza sativa L.). Bioscience reports **27**, 225-234.

Liu, Z., Zhu, Y., Gao, J., Yu, F., Dong, A., and Shen, W.H. (2009). Molecular and reverse genetic characterization of NUCLEOSOME ASSEMBLY PROTEIN1 (NAP1) genes unravels their function in transcription and nucleotide excision repair in Arabidopsis thaliana. Plant J **59,** 27-38.

Luo, Q., Wang, B., Wu, Z., Jiang, W., Wang, Y., Du, K., Zhou, N., Zheng, L., Gan, J., Shen, W.H., Ma, J., and Dong, A. (2020). NAP1-Related Protein 1 (NRP1) has multiple interaction modes for chaperoning histones H2A-H2B. Proc Natl Acad Sci U S A **117,** 30391-30399.
