# Supplementary material for: The chromatin remodeler ERCC6 and the histone chaperone NAP1 are involved in apurinic/apyrimidinic endonuclease-mediated DNA repair
Source: Plant Cell. 2024 Feb 17;36(6):2238–52. doi: 10.1093/plcell/koae052 (PMC11132878; doi:10.1093/plcell/koae052)
Supplement: koae052_Supplementary_Data [file tpc.01027.2023-s10.pdf › tpc.01027.2023-s10.pdf]

THE PLANT CELL

Involvement of chromatin remodeler ERCC6 and histone chaperone NAP1 in apurinic/apyrimidinic endonuclease-mediated DNA repair

Tianyi Fan, Tianfang Shi, Ran Sui, Huijia Kang, Yao Yu, Jingqi Wang, and Yan ZHU

Review Timeline:

Submission Date:	31-Oct-2023
Editorial Decision:	21-Nov-2023
Revision Received:	10-Jan-2024
Editorial Decision:	24-Jan-2024
Revision Received:	26-Jan-2024
Accepted:	28-Jan-2024

Prof. Dr. Yan ZHU
School of life sciences, Fudan University
Shanghai 200433
China

Dear Dr. ZHU:

We have received reviews of your manuscript entitled "Involvement of chromatin remodeler ERCC6 and histone chaperone NAP1 in apurinic/apyrimidinic endonuclease-mediated DNA repair." Thank you for submitting your best work to The Plant Cell. The editorial board agrees that the work you describe is substantive, falls within the scope of the journal, and may become acceptable for publication, pending revision and potential re-review.

We ask you to pay attention to all comments made by the expert reviewers in preparing your revision, but especially the following points. The reviewers indicated that additional controls and assays are needed to validate the interaction between ARP, CHR24/ERCC6 and NAP1. There are also genetic and biochemical experiments suggested that would help validate the proposed role of CHR24/ERCC6 and NAP1 in facilitating BER. The ideal experiment would be an in vitro BER assay using chromatin templates and ARP alone or in complex with CHR24/ERCC6 and/or NAP1. In this in vitro assay, point mutants of individual components that cannot interact could also be tested. If not technically possible, then providing additional genetic evidence as suggested by the reviewers would become very important.

Please contact us if there are ambiguous comments or if you wish to discuss the revision.

Given the nature of the comments, we are offering you 60 days from when we have issued this decision to complete the revision. If you think you will need more than 60 days please let us know. If a revision is not returned within this time frame, and if you have not been granted an extension, we may withdraw the manuscript, which would leave you free to submit the work elsewhere. If you need an extension, we encourage you to contact us at any point before the 60 days have passed.

When you are ready to submit the revised version, please upload a highlighted copy that indicates all changes made in response to the editor and reviewer recommendations. Include an itemized list of all changes made in response to each of the reviewer's suggestions in the "Response to Reviewers" section; please note that reviewers do not have access to your cover letter, nor was this decision letter shared with them.

Thank you for the privilege of reviewing your work. We look forward to receiving your revised manuscript.

Sincerely,

The Plant Cell Editorial Board

Please note the following:

-The Plant Cell now requires authors to complete and submit an author revisions checklist upon submission of a revised manuscript. The aim of the checklist is to aid authors in preparing a high-quality manuscript, facilitate the review and assessment of revised manuscripts, and help to ensure that journal standards are maintained across the board. If your manuscript is accepted, the completed checklist will be published as supplemental material attached to the article online. Please download a copy of the checklist (pdf fillable form) at this link, for submission with your revised manuscript: https://tpc.msubmit.net/html/Author_Revisions_Checklist.pdf.

-Supplemental materials should be restricted to large datasets and tables, presentation of replicates, and validation of reagents, methods, or genotypes. Any data that are used to support the major claims must be in the main manuscript. Supplemental figure legends must indicate what figure in the main manuscript is supported by the supplemental data presented. Please justify how each of the supplemental figures meet the criteria.

-Sampling methods and nature of "biological replicates" should be described precisely (i.e. different plants, parts of plants, pooled tissue, independent pools of tissue, sampled at different times, etc), along with a clear description of and rationale for any statistical analyses conducted. The reader should know exactly what was sampled; what forms the basis of the calculation of any means and statistical parameters reported. This is also necessary to ensure that proper statistical analysis was conducted.

-Want to add this revision deadline to your calendar? Click below!

-- Reviewer comments:

Reviewer #1 (Comments for the Author):

This study identified APE-redox protein (ARP)-interacting proteins by pull down combined with MS, and showed that a chromatin remodeling protein, CHR24, and a histone chaperone, NAP1;2 is ARP-interacting proteins. The authors also showed some data and tried to demonstrate that CHR24 and its homologs CHR8 redundantly function together with ARP and NAP1;2 to regulate the base excision repair. However, I feel that several data shown in the current study are preliminary and need to be validated by further experiments. Without further experiments, the function of CHR24 and CHR8 in ARP-mediated base excision repair cannot be concluded.

1. Although CHR24 and NAP1;2 were co-purified with ARP by pull down-MS assay, the interactions need to be confirmed by in vivo assays using stably expressed CHR24 and NAP1;2 transgenic plants. In the current study, the interactions were validated either by in vitro assays or by using transiently expressed protoplasts.

2. Because the transgenic plants expressing ENL1, an ortholog of CHR24, have been detectable in rice, it is surprising that the stably expressed CHR24 transgenic lines cannot be obtained in Arabidopsis plants. CHR24 and NAP1;2 transgenic plants need to be obtained to confirm the interactions between ARP and CHR24 and between ARP and NAP1;2.

3. In Figure 1E, the interactions of CHR24 with NAP1;2 and ARP were detected by pull down assays. The GST-CHR24-N and CHR24-C also showed a weak interaction with ARP, as indicated on the MYC-ARP blots. Moreover, the interaction between each part of CHR24 and ARP is very weak, challenging the specificity of these bindings.

4. The ATPase activity and nucleosome remodeling activity were demonstrated for CHR24 and NAP1;2. However, this result is not an evidence for supporting that CHR24 and NAP1;2 are involved in ARP-mediated base excision repair. It is not surprising that a chromatin remodeling protein is involved in enhancing the access of DNA.

5. Although the double ercc6 mutant was shown to be sensitive to 5-FU, the phenotype was not tested by complementation testing. It is possible that the phenotype was caused by other unidentified mutations.

6. The chr24-2 mutant was identified in the study but was not used for detecting its effect on 5-FU sensitivity.

7. The yeast ARP, NAP1, and ERCC6 homologs were co-expressed in yeast strains and were used for detecting the interaction of these proteins. The result shown in Figure 4A indicated that these proteins seem to interact with each other in yeast. However, the interactions are weak based on the result, and proper negative controls are required for the experiment, such as unrelated tagged proteins. I feel that it is more appropriate to detect the interacting proteins of ARP, NAP1, and ERCC6 homologs by pull-down combined with MS in yeast cells.

8. In Figure 4B, the result did not support the conserved role of ERCC6 homologs in base excision repair.

Reviewer #2 (Comments for the Author):

This is a review of the manuscript „Involvement of chromatin remodeler ERCC6 and histone chaperone NAP1 in apurinic/apyrimidinic endonuclease-mediated DNA repair" by Fan and colleagues submitted to the Plant Cell. This study focused on the mechanism of base excision repair in plants. The main novelty is presented by associating specific BER pathways with the DNA damage repair in the chromatin context. Using a mix of genetic and biochemical methods, the authors show that Swi/Snf2 type chromatin remodeler ERCC6 and histone chaperone NAP1 take place in BER. The interaction discovered in Arabidopsis was validated also in yeast.
This is a very timely and original work that brings novel insight into the mechanism of BER. It will be of interest to both plant DNA damage repair and chromatin communities.
I have several points which would improve the manuscript.
1. Plant gene/protein writing style locally does not follow the rules used in the Plant Cell or the gene full names are missing (e.g. lines 73-75).
2. Lines 149-152: I think that this part should be supported by data. In particular when it conflicts with previously published data.

3. Lines from 154. Please specify the sizes of the tagged proteins in the text.
4. Line 166: Remove the word „significantly".
5. Line 183: How do the authors know that the defective protein function is due to lack of ATPase activity and not for example protein conformational changes induced by the exchanged amino acid sequence?
6. Line 274: I wonder why the selection was done in F3 generation when the easier way would be in F2. Or was an F2 plant of specific genotype selected first and then the F3 progeny was screened? This has an impact on the expected frequencies.
7. Line 281: Please explain the statement „suggesting that plant growth is sensitive to the ERCC6 gene copy". I am not sure what is the meaning of this sentence.
8. Line 312: Delete the statement „the simplest unicellular eukaryote" as it is not true.
9. Images of Western blots: Please consider adding arrows to the position of the expected position of the band. Some WBs have multiple bands and I get confused about which one is correct.
10. Figure 1B legend: Please add more information on what the figure shows.
11. Figure 2D: Hrs is probably missing in the figure.
12. Figure 3A: The bootstrap values are too small.
13. Figure 3C+D, corresponding figure legends: Please consider replacing the term „wet weight" with „fresh weight" which is a more standard form.
14. Figure 3C legend: Add information on the number of plants in each biological replicate and information on which statistical test was applied.
15. Figure 3D legend: Add information on what error bars represent.

Reviewer #3 (Comments for the Author):

Apurinic or apyrimidinic (AP) site is the intermediate product in base excision repair (BER). The endonuclease ARP cleaved AP sites, which is essential for successful BER. Chromatin structure has been shown to affect the DNA repair process. However, how chromatin remodeling contributes to BER is largely unknown. In this manuscript, the authors found that ARP interacts with chromatin remodeler CHR24 and histone chaperone NAP1 in Arabidopsis. They proposed that CHR24 and NAP1 are involved in BER. Finally, they showed that this mechanism is conserved in yeasts. Therefore, this study provides new insights into BER. I have the following concerns.

1. The relationship between ARP and CHR24/NAP1 needs to be strengthened. Although the authors showed that these proteins interact with each other and the arp, arp chr24 and arp nap1-tri mutants showed similar sensitivity to 5-FU, there is no direct evidence showing that CHR24 and NAP1 facilitated ARP's function in BER. The hypersensitivity of the chr24 and nap1-tri mutants to 5-FU supports the involvement of CHR24 and NAP1 in BER. But they may function independently of ARP. Biochemical experiments, in which the cleavage activity of ARP is enhanced by CHR24/NAP1, should be performed. Furthermore, the authors can test whether overexpression of CHR24/NAP1 suppresses the arp mutant or whether overexpression of ARP suppresses the chr24 or nap1 mutants. It is noted that there are two other AP endonucleases, APE2 and APE1L, in Arabidopsis.
2. The interaction between APR and CHR24/NAP1 needs to be further clarified. In Fig. 1A, the "no Ab" control is not sufficient to demonstrate the interactions are specific. The protein tags (YFP, MYC, FLAG) could contribute to the interaction. To exclude this possibility, the protein tags alone or fused with other proteins should be used as negative controls. Fig. 4A has similar issues. In Fig. 1E, according to the upper panel, in the GST-CHR24-ATPase sample, there was a lot of GST protein. Therefore, the total amount of GST-CHR24-ATPase + GST was much more than that of the GST control, which made the result unconvincing. The authors should increase the GST amount in the pull-down assay. The used CoIP and pull-down assays to test protein interactions. However, both assays are subjected to variations depending on the experiment condition. Alternative assays such as Y2h, BiFC, or split luciferase could be used to confirm the protein interactions.

Response to *Reviewers*

Reviewer #1 (Comments for the Author):

This study identified APE-redox protein (ARP)-interacting proteins by pull down combined with MS, and showed that a chromatin remodeling protein, CHR24, and a histone chaperone, NAP1;2 is ARP-interacting proteins. The authors also showed some data and tried to demonstrate that CHR24 and its homologs CHR8 redundantly function together with ARP and NAP1;2 to regulate the base excision repair. However, I feel that several data shown in the current study are preliminary and need to be validated by further experiments. Without further experiments, the function of CHR24 and CHR8 in ARP-mediated base excision repair cannot be concluded.

Response: We thank Reviewer 1 for the suggestions.

1. Although CHR24 and NAP1;2 were co-purified with ARP by pull down-MS assay, the interactions need to be confirmed by in vivo assays using stably expressed CHR24 and NAP1;2 transgenic plants. In the current study, the interactions were validated either by in vitro assays or by using transiently expressed protoplasts.

2. Because the transgenic plants expressing ENL1, an ortholog of CHR24, have been detectable in rice, it is surprising that the stably expressed CHR24 transgenic lines cannot be obtained in Arabidopsis plants. CHR24 and NAP1;2 transgenic plants need to be obtained to confirm the interactions between ARP and CHR24 and between ARP and NAP1;2.

Response: We thank *Reviewer 1* for the suggestion. In this study, we encountered challenges in obtaining stable transgenic Arabidopsis alleles expressing CHR24 or ARP protein. It is reasonable that ectopic expression of certain catalytic activities throughout plant growth and development may potentially induce toxicity.

Although ENL1 is considered as the CHR24 ortholog in rice, it has been demonstrated that loss-of-function mutations in Arabidopsis *CHR24* and rice *ENL1* have distinct effects on endosperm

nuclei and normal plant growth (**Hara et al., 2015**). Notably, Arabidopsis CHR24 exhibits limited protein similarity to rice ENL1 in their ATPase domains and C-termini, but significantly lower protein similarity in their N-termini (see **Supplementary Information 1** below), suggesting potential differences in their protein interactomes and biological roles.

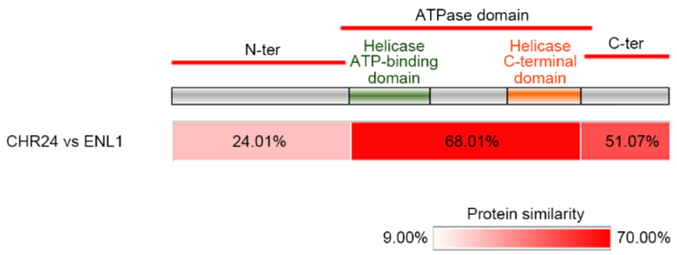

Supplementary Information 1. The protein similarity between Arabidopsis CHR24 and rice ENL1.

Supplementary Reference

Hara, T., Katoh, H., Ogawa, D., Kagaya, Y., Sato, Y., Kitano, H., Nagato, Y., Ishikawa, R., Ono, A., Kinoshita, T., Takeda, S., and Hattori, T. (2015). Rice SNF2 family helicase ENL1 is essential for syncytial endosperm development. Plant J **81,** 1-12.

3. In Figure 1E, the interactions of CHR24 with NAP1;2 and ARP were detected by pull down assays. The GST-CHR24-N and CHR24-C also showed a weak interaction with ARP, as indicated on the MYC-ARP blots. Moreover, the interaction between each part of CHR24 and ARP is very weak, challenging the specificity of these bindings.

Response: We thank *Reviewer 1* for the suggestion. In response, we have conducted a repeated pulldown assay to validate the specificity of these bindings (Figure 1E). We sincerely hope that our efforts will meet the expectations of *Reviewer* 1.

4. The ATPase activity and nucleosome remodeling activity were demonstrated for CHR24 and NAP1;2. However, this result is not an evidence for supporting that CHR24 and NAP1;2 are involved in ARP-mediated base excision repair. It is not surprising that a chromatin remodeling protein is involved

in enhancing the access of DNA.

Response: Our protein examinations, including IP-MS (Supplemental Table S1), pulldown (Figure 1C, 1E, Supplemental Figure 3), and co-IP assays (Figure 1A, Supplemental Figure 2), have unequivocally demonstrated the specific interaction between ARP and CHR24 as well as NAP1;2. Furthermore, our genetic analysis has substantiated the involvement of *CHR24* (but not another chromatin remodeler gene *INO80*) and *NAP1* (but not its close related chaperone genes *NRP*) in ARP-mediated base excision repair (Supplemental Figure 9). Although it is not unexpected for a chromatin remodeling factor or histone chaperone to be recruited to enhance local DNA accessibility, our findings provide compelling evidence for the specificity of such recruitment.

5. Although the double ercc6 mutant was shown to be sensitive to 5-FU, the phenotype was not tested by complementation testing. It is possible that the phenotype was caused by other unidentified mutations.

6. The chr24-2 mutant was identified in the study but was not used for detecting its effect on 5-FU sensitivity.

Response: We have demonstrated complementation testing in the first manuscript (original Supplemental Figure 7; now Supplemental Figure 9 in the revised manuscript), which provides evidence that the *ercc6* double mutant phenotype is attributed to functional disruption of *CHR8/CHR24*. In the revised manuscript, we have included additional phenotype analysis of *chr24-2* in Supplemental Figure 8.

7. The yeast ARP, NAP1, and ERCC6 homologs were co-expressed in yeast strains and were used for detecting the interaction of these proteins. The result shown in Figure 4A indicated that these proteins seem to interact with each other in yeast. However, the interactions are weak based on the result, and proper negative controls are required for the experiment, such as unrelated tagged proteins. I feel that it is more appropriate to detect the interacting proteins of ARP, NAP1, and ERCC6 homologs by pull-down combined with MS in yeast cells.

Response: We express our gratitude to *Reviewer 1* for the valuable suggestion. We constructed two expression vectors MYC-GFP and FLAG-mCherry as the negative controls for the tagged proteins. In yeast cells, we co-expressed Nap1-MYC+FLAG-Apn1 (with MYC-GFP+FLAG-Apn1 as the negative control); Nap1-MYC+HA-Rad26 (with MYC-GFP+HA-Rad26 as the negative control); and FLAG-Apn1+HA-Rad26 (with FLAG-mCherry+HA-Rad26 as the negative control). Our findings are now illustrated in Supplemental Figure 14 of the revised manuscript, demonstrating specific binding interactions among Nap1-MYC, FLAG-Apn1, and HA-Rad26.

8. In Figure 4B, the result did not support the conserved role of ERCC6 homologs in base excision repair.

Response: We have demonstrated that the *rad26*Δ strain displays similar sensitivity as *apn1*Δ strain to the alkylating agent MMS, which can also trigger base excision repair (Supplemental Figure 15). Unlike 5-FU, MMS triggers UNG-independent base excision repair. In our manuscript, we have extensively discussed the distinct contributions of yeast Nap1 and Rad26 in the Apn1-mediated base excision repair triggered by different types of base lesions.

Reviewer #2 (Comments for the Author):

This is a review of the manuscript „Involvement of chromatin remodeler ERCC6 and histone chaperone NAP1 in apurinic/apyrimidinic endonuclease-mediated DNA repair" by Fan and colleagues submitted to the Plant Cell. This study focused on the mechanism of base excision repair in plants. The main novelty is presented by associating specific BER pathways with the DNA damage repair in the chromatin context. Using a mix of genetic and biochemical methods, the authors show that Swi/Snf2 type chromatin remodeler ERCC6 and histone chaperone NAP1 take place in BER. The interaction discovered in Arabidopsis was validated also in yeast.

This is a very timely and original work that brings novel insight into the mechanism of BER. It will be of interest to both plant DNA damage repair and chromatin communities.

Response: We thank *Reviewer 2* for the appreciation.

I have several points which would improve the manuscript.

1. Plant gene/protein writing style locally does not follow the rules used in the Plant Cell or the gene full names are missing (e.g. lines 73-75).

2. Lines 149-152: I think that this part should be supported by data. In particular when it conflicts with previously published data.

3. Lines from 154. Please specify the sizes of the tagged proteins in the text.

4. Line 166: Remove the word „significantly".

Response: We thank *Reviewer 2* for the suggestions. We have corrected the texts in the revised manuscript.

5. Line 183: How do the authors know that the defective protein function is due to lack of ATPase activity and not for example protein conformational changes induced by the exchanged amino acid sequence?

Response: We mutated the key residues (GLGKT→DEDEV) within the ATPase domain (474 aa-long), which are specifically involved in binding ATP phosphate. The remaining protein sequence (469 aa-long) and overall protein sequence length were kept unaltered. Structural modeling analysis of both CHR24-ATPase and CHR24-ATPase^{mut} domains were conducted and compared using SWISS-MODEL (see **Supplementary Information 2** below). The modeling results indicated that the GLGKT motif is situated in a relatively flexible region, potentially associated with its dynamic interaction with ATP phosphate. However, mutation of these residue does not impact the overall conformation of this domain, as both domains are predicted to exhibit nearly identical conformation. We acknowledge that these findings are based solely on model- and AI-based prediction without further experimental validation through protein crystallization. Therefore, we cannot completely exclude the possibility of any major conformational changes occurring in the entire protein structure due to these amino acid substitutions. We have changed our expression in the revised manuscript.

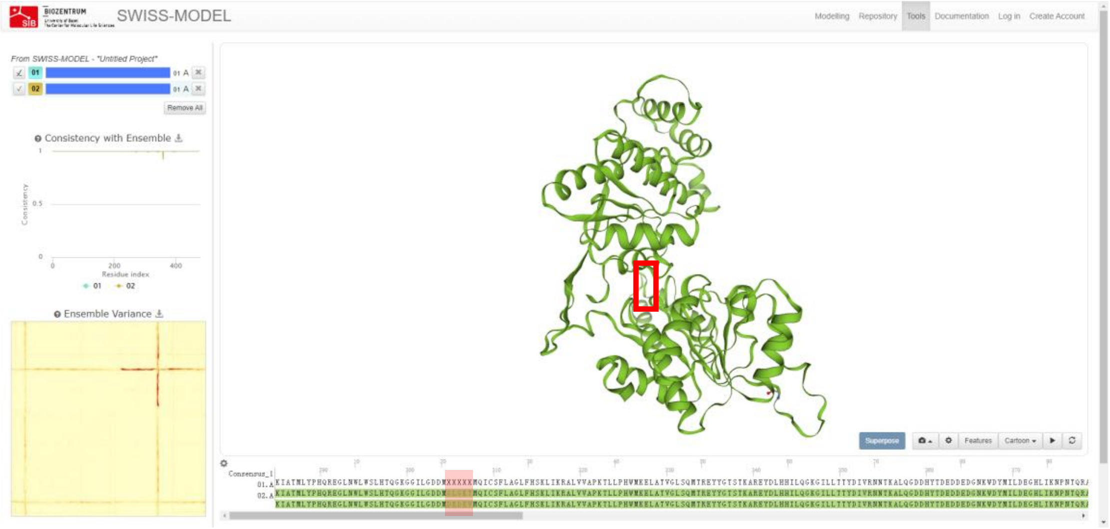

Supplementary Information 2. The predicted protein conformation of CHR24-ATPase and CHR24-ATPase^{mut}.

The protein conformation of CHR24-ATPase and CHR24-ATPase^{mut} were modeled and compared using SWISS-MODEL (https://swissmodel.expasy.org/interactive). Both domains are predicted to exhibit nearly identical conformation. The red box indicates the predicted location of GLGKT motif, and the light red squared highlight the mutated residues (GLGKT→DEDEV).

6. Line 274: I wonder why the selection was done in F3 generation when the easier way would be in F2. Or was an F2 plant of specific genotype selected first and then the F3 progeny was screened? This has an impact on the expected frequencies.

Response: We obtained *ercc6 nap1;1/3* and *chr24 nap1-tri* quadruple mutants in the F2 generation. However, our attempts to isolate quintuple mutants from subsequent generations of *ercc6 nap1;1/3* were ultimately unsuccessful.

7. Line 281: Please explain the statement „suggesting that plant growth is sensitive to the ERCC6 gene copy". I am not sure what is the meaning of this sentence.

Response: We apologize for the inaccuracies present in the text and have made necessary revisions to our statement in the revised manuscript.

8. Line 312: Delete the statement „the simplest unicellular eukaryote" as it is not true.

9. Images of Western blots: Please consider adding arrows to the position of the expected position of the band. Some WBs have multiple bands and I get confused about which one is correct.

10. Figure 1B legend: Please add more information on what the figure shows.

11. Figure 2D: Hrs is probably missing in the figure.

12. Figure 3A: The bootstrap values are too small.

13. Figure 3C+D, corresponding figure legends: Please consider replacing the term „wet weight" with „fresh weight" which is a more standard form.

14. Figure 3C legend: Add information on the number of plants in each biological replicate and information on which statistical test was applied.

15. Figure 3D legend: Add information on what error bars represent.

Response: The suggestions provided by *Reviewer 2* are greatly appreciated. The texts and figures in the revised manuscript have been appropriately rectified.

Reviewer #3 (Comments for the Author):

Apurinic or apyrimidinic (AP) site is the intermediate product in base excision repair (BER). The endonuclease ARP cleaved AP sites, which is essential for successful BER. Chromatin structure has been shown to affect the DNA repair process. However, how chromatin remodeling contributes to BER is largely unknown. In this manuscript, the authors found that ARP interacts with chromatin remodeler CHR24 and histone chaperone NAP1 in Arabidopsis. They proposed that CHR24 and NAP1 are involved in BER. Finally, they showed that this mechanism is conserved in yeasts. Therefore, this study provides new insights into BER.

Response: We thank *Reviewer 3* for the appreciation.

I have the following concerns.

1. The relationship between ARP and CHR24/NAP1 needs to be strengthened. Although the authors showed that these proteins interact with each other and the arp, arp chr24 and arp nap1-tri mutants showed similar sensitivity to 5-FU, there is no direct evidence showing that CHR24 and NAP1 facilitated ARP's function in BER. The hypersensitivity of the chr24 and nap1-tri mutants to 5-FU supports the involvement of CHR24 and NAP1 in BER. But they may function independently of ARP. Biochemical experiments, in which the cleavage activity of ARP is enhanced by CHR24/NAP1, should be performed. Furthermore, the authors can test whether overexpression of CHR24/NAP1 suppresses the arp mutant or whether overexpression of ARP suppresses the chr24 or nap1 mutants. It is noted that there are two other AP endonucleases, APE2 and APE1L, in Arabidopsis.

Response: We express our gratitude to *Reviewer 3* for the valuable suggestions.

During the revision process, we performed nucleosome reconstitution using 225 bp-long AP-containing DNA and recombinant histone octamers (revised Figure 2C). Our *in vitro* experiments demonstrated that MBP-ARP alone exhibits clear endonuclease activity towards naked DNA; however, the nucleosomal structure significantly impedes its cleavage at AP sites. Notably, GST-CHR24 specifically enhances the endonuclease activity of MBP-ARP towards nucleosomes rather than naked DNA in an ATP-dependent manner and in synergy with His-NAP1;2. The updated findings have been

presented in revised Figure 2F.

As stated in the manuscript, we encountered challenges in obtaining transgenic Arabidopsis alleles highly expressing CHR24 or ARP protein during this study. We postulate that this could be attributed to potential toxicity associated with ectopic expression of these catalytic activities throughout plant growth and development. Consequently, we regret our inability to provide a satisfactory response to the reviewer's speculative inquiry regarding genetic suppression by overexpression of CHR24/ARP.

Despite the presence of two other AP endonucleases, APE2 and APE1L, in Arabidopsis, ARP (APE-Redox Protein) has been established as the predominant enzyme responsible for cleaving apurinic/apyrimidinic (AP) sites (**Cordoba-Canero et al., 2009; Cordoba-Canero et al., 2011; Li et al., 2023**).

Supplementary Reference

Cordoba-Canero, D., Roldan-Arjona, T., and Ariza, R.R. (2011). Arabidopsis ARP endonuclease functions in a branched base excision DNA repair pathway completed by LIG1. Plant J **68,** 693-702.

Cordoba-Canero, D., Morales-Ruiz, T., Roldan-Arjona, T., and Ariza, R.R. (2009). Single-nucleotide and long-patch base excision repair of DNA damage in plants. Plant J **60,** 716-728.

Li, J., Wang, C., Liang, W., Zhang, J., Jiang, C.K., Liu, Y., Ren, Z., Ci, D., Chang, J., Han, S., Deng, X.W., Wang, Y., and Qian, W. (2023). Functional importance and divergence of plant apurinic/apyrimidinic endonucleases in somatic and meiotic DNA repair. Plant Cell **35,** 2316-2331.

2. The interaction between APR and CHR24/NAP1 needs to be further clarified. In Fig. 1A, the "no Ab" control is not sufficient to demonstrate the interactions are specific. The protein tags (YFP, MYC, FLAG) could contribute to the interaction. To exclude this possibility, the protein tags alone or fused

with other proteins should be used as negative controls. Fig. 4A has similar issues. In Fig. 1E, according to the upper panel, in the GST-CHR24-ATPase sample, there was a lot of GST protein. Therefore, the total amount of GST-CHR24-ATPase + GST was much more than that of the GST control, which made the result unconvincing. The authors should increase the GST amount in the pull-down assay. They used CoIP and pull-down assays to test protein interactions. However, both assays are subjected to variations depending on the experiment condition. Alternative assays such as Y2h, BiFC, or split luciferase could be used to confirm the protein interactions.

Response: We express our gratitude to *Reviewer 3* for the valuable suggestions. During the revision process, we performed the co-IP in Arabidopsis mesophyll protoplasts (revised Supplemental Figure 2) and yeast cells (revised Supplemental Figure 14) by co-expressing two proteins (or controls) with different tags. The complete set of combinations is provided as **Supplementary Information 3** below.

Arabidopsis mesophyll protoplasts				
**Co-expression**	**Clone 1**	**Clone 2**	**IP**	**Western blot**
**Experiment 1**	MYC-ARP	CHR24-FLAG	@MYC	@MYC, @FLAG
**Control 1**	MYC-ARP	RAD51-FLAG	@MYC	@MYC, @FLAG
**Experiment 2**	YFP-NAP1;2	CHR24-FLAG	@GFP	@GFP, @FLAG
**Control 2**	YFP-NAP1;2	RAD51-FLAG	@GFP	@GFP, @FLAG
**Experiment 3**	MYC-ARP	YFP-NAP1;2	@MYC	@MYC, @GFP
**Control 3**	MYC-ARP	YFP-RRM	@MYC	@MYC, @GFP

Yeast cells				
**Co-expression**	**Clone 1**	**Clone 2**	**IP**	**Western blot**
**Experiment 1**	Nap1-MYC	FLAG-Apn1	@MYC	@MYC, @FLAG
**Control 1**	MYC-GFP	FLAG-Apn1	@MYC	@MYC, @FLAG
**Experiment 2**	Nap1-MYC	HA-Rad26	@MYC	@MYC, @HA
**Control 2**	MYC-GFP	HA-Rad26	@MYC	@MYC, @HA
**Experiment 3**	FLAG-Apn1	HA-Rad26	@FLAG	@FLAG, @HA
**Control 3**	FLAG-mCherry	HA-Rad26	@FLAG	@FLAG, @HA

Supplementary Information 3. All the co-expressed proteins in Arabidopsis mesophyll protoplasts (upper table) and yeast cells (lower table) are shown.

RAD51-FLAG (**Fan et al., 2022**) serves as the control for CHR24-FLAG. Overexpression of r*FCA* RRM (RNA recognition motifs) has been shown to regulate cellular size in rice (**Hong et al., 2007**). We cloned the close homolog of functional RRM from Arabidopsis *FCA* gene and expressed it as a YFP-tagged protein, serving as a control for YFP-NAP1;2. Besides, we constructed two clones expressing MYC-GFP and FLAG-mCherry as controls in yeast cells.

In the revision stage, we repeated the pulldown experiment depicted in revised Figure 1E. The

control GST amount has been significantly increased in the pulldown assay. Again, our findings demonstrated that CHR24-ATPase can effectively pull down both YFP-NAP1;2 and MYC-ARP, and at the same time, compared to its native form, GST-CHR24-ATPase^{mut} exhibited reduced affinity.

We sincerely hope that our endeavors will meet the expectations of *Reviewer 3*.

Supplementary Reference

Fan, T., Kang, H., Wu, D., Zhu, X., Huang, L., Wu, J., and Zhu, Y. (2022). Arabidopsis gamma-H2A.X-INTERACTING PROTEIN participates in DNA damage response and safeguards chromatin stability. Nature communications **13,** 7942.

Hong, F., Attia, K., Wei, C., Li, K., He, G., Su, W., Zhang, Q., Qian, X., and Yang, J. (2007). Overexpression of the rFCA RNA recognition motif affects morphologies modifications in rice (Oryza sativa L.). Bioscience reports **27,** 225-234.

Dear Dr. Yan Zhu,

We have received reviews of your manuscript entitled "Involvement of chromatin remodeler ERCC6 and histone chaperone NAP1 in apurinic/apyrimidinic endonuclease-mediated DNA repair." On the basis of the advice received, the board of reviewing editors would like to accept your manuscript for publication in The Plant Cell. This acceptance is contingent on one revision based on a comment from one of our reviewers.

In the legend for Supplemental Figure S8B, the asterisk was not shown in the figure but was mentioned in the legend. If there is no significant difference between two samples, NS should be indicated in the figure and in the legend.

Please highlight all changes and include a detailed annotation of changes of the text, with line numbers, and noting your responses to the comments.

To submit your revised manuscript, click:

Link Not Available

If you have any questions about the revision submission procedures, please contact the Editorial Office Staff (tpc-submissions@aspb.org). If you cannot return the revised manuscript within 30 days, please let us know. Otherwise, we will assume that you have elected not to revise the manuscript and withdraw it.

Thank you very much for the privilege of reviewing this work. I look forward to receiving the next version.

Sincerely,

The Plant Cell Editorial Board

Reviewer #1 (Comments for the Author):

Most of my concerns raised on the first version manuscript have been addressed in the current manuscript. One remaining concern (mentioned in point 1) is that the interaction between CHR24 and NAP1;2 was not detected by using stable transgenic plants. The authors have explained that the stable transgenic plants expressing CHR24 or ARP are not available. Considering the interaction between CHR24 and NAP1;2 has been supported by other evidences, I feel that this explanation is acceptable. Moreover, I have a minor suggestion. In the legend for Supplemental Figure S8B, Asterisk was not shown in in the figure but was mentioned in the legend. If there is no significant difference between two samples, NS should be indicated in the figure and in the legend.

Reviewer #2 (Comments for the Author):

The authors have successfully addressed all my concerns and performed the requested/suggested changes in the text.

Reviewer #3 (Comments for the Author):

The authors have performed addition experiments to support the conclusion. I have no other concerns.

Please also note the following:

-The Plant Cell now requires authors to complete and submit an author revisions checklist upon submission of a revised

manuscript. The aim of the checklist is to aid authors in preparing a high-quality manuscript, facilitate the review and assessment of revised manuscripts, and help to ensure that journal standards are maintained across the board. If your manuscript is accepted, the completed checklist will be published as supplemental material attached to the article online. Please download a copy of the checklist (pdf fillable form) at this link, for submission with your revised manuscript: https://tpc.msubmit.net/html/Author_Revisions_Checklist.pdf.

-Supplemental materials should be restricted to large datasets and tables, presentation of replicates, and validation of reagents, methods, or genotypes. Any data that are used to support the major claims must be in the main manuscript. Supplemental figure legends must indicate what figure in the main manuscript is supported by the supplemental data presented. Please justify how each of the supplemental figures meet the criteria.

-Sampling methods and the nature of "biological replicates" should be described precisely (i.e. different plants, parts of plants, pooled tissue, independent pools of tissue, sampled at different times, etc.), along with a clear description of and rationale for any statistical analyses conducted. The reader should know exactly what was sampled; what forms the basis of the calculation of any means and statistical parameters reported. This is also necessary to ensure that proper statistical analysis was conducted.

Response to *Reviewers*

Reviewer #1 (Comments for the Author):

Most of my concerns raised on the first version manuscript have been addressed in the current manuscript. One remaining concern (mentioned in point 1) is that the interaction between CHR24 and NAP1;2 was not detected by using stable transgenic plants. The authors have explained that the stable transgenic plants expressing CHR24 or ARP are not available. Considering the interaction between CHR24 and NAP1;2 has been supported by other evidences, I feel that this explanation is acceptable. Moreover, I have a minor suggestion. In the legend for Supplemental Figure S8B, Asterisk was not shown in in the figure but was mentioned in the legend. If there is no significant difference between two samples, NS should be indicated in the figure and in the legend.

Response: We thank *Reviewer 1* for the appreciation. We are sorry for the editorial mistake in the figure legend for *Supplemental Figure S8B* during manuscript preparation. The comparison between the wild type (WT) and mutant did not yield any statistically significant differences (P = 0.415, *t*-test, two-tail). Here, we corrected our statement in the figure legend for S8B in the revised "Supplementary information".

Reviewer #2 (Comments for the Author):

The authors have successfully addressed all my concerns and performed the requested/suggested changes in the text.

Response: We thank *Reviewer 2* for the appreciation.

Reviewer #3 (Comments for the Author):

The authors have performed addition experiments to support the conclusion. I have no other concerns.

Response: We thank *Reviewer 3* for the appreciation.

Dear Dr. Yan ZHU:

We are pleased to inform you that your paper entitled "Involvement of chromatin remodeler ERCC6 and histone chaperone NAP1 in apurinic/apyrimidinic endonuclease-mediated DNA repair" has been accepted for publication in The Plant Cell, pending a final editorial review by a science editor. At this stage, your manuscript will be evaluated by a science editor with respect to its presentation of scientific content, compliance with journal policies, and presentation for a broad readership. The Plant Cell has contracted with Plant Editors (planteditors.com) to provide this service to our authors, and you will soon receive additional information on this process.

Please note that each author needs to link their ORCID identifier to their account in the system before your manuscript can be published. If any authors do not have an ORCID linked to their account, they will receive a message with a link to complete this task. Please ensure that ALL of your coauthors have completed this task as soon as possible.

ASPB offers an OPEN option that allows authors to have their online articles available for free to all users immediately upon publication. For more information about the ASPB OPEN option, refer to the Final Submission Checklist Form.

The Plant Cell and The Arabidopsis Information Resource (TAIR) are collaborating to collect functional annotation data about Arabidopsis genes from authors. This includes information about the gene's molecular function (e.g., kinase activity, ATP synthetase activity), the biological process/es it is involved in (e.g., endosperm development, threonine biosynthesis), its subcellular location (e.g., nucleus, ER), anatomical or developmental expression pattern (e.g., leaf, ovule, flower stage 10, seedling stage), or its partner in a protein-protein interaction (e.g., AT1G01010 interacts with AT1G01020).

If your paper contains results falling into one or more of these categories for Arabidopsis genes, we request that you now submit these data for inclusion in TAIR by filling in the form provided at the following URL: https://www.arabidopsis.org/doc/submit/functional_annotation/123. If you need further clarification on what types of data can be submitted please contact curator@arabidopsis.org.

Finally, we encourage your submission of artwork for the journal cover. Monthly issues will have an online cover image and selected covers will be used for posters, other promotional items, and "wallpaper" for mobile devices. For more information, refer to Cover Submission in the Instructions for Authors https://tpc.msubmit.net/cgi-bin/main.plex?form_type=display_auth_instructions.

We look forward to seeing your paper published.

Sincerely,

The Plant Cell Editorial Board

=====
IMPORTANT REMINDER: PEER REVIEW REPORTS
=====

If you opted to publish a peer review report along with your article during the original submission process, it will be prepared by the editorial staff and publicly posted with your manuscript, inside the zip file that contains any other supplemental material. As a reminder, the peer review report is a public record of all comments from editors and reviewers, as well as your prior responses, as you received them in the decision letters for each draft of your manuscript. If you agreed to publish this report and have changed your mind, or are not sure if you selected this option, please contact the editorial office as soon as possible before signing the license agreement from our publisher.

=====

----FOR ASPB OFFICE USE ONLY (DO NOT EDIT)----
MSID: 36824
Corresponding Author: Prof. Dr. ZHU
